# A translational synthetic biology platform for rapid access to gram-scale quantities of novel drug-like molecules

**DOI:** 10.1016/j.ymben.2017.06.012

**Published:** 2017-07

**Authors:** James Reed, Michael J. Stephenson, Karel Miettinen, Bastiaan Brouwer, Aymeric Leveau, Paul Brett, Rebecca J.M. Goss, Alain Goossens, Maria A. O’Connell, Anne Osbourn

**Affiliations:** a Department of Metabolic Biology, John Innes Centre, Norwich Research Park, Norwich NR4 7UH, UK; b Department of Plant Systems Biology, VIB, Ghent University, B-9052 Gent, Belgium; c Department of Plant Biotechnology and Bioinformatics, Ghent University, B-9052 Gent, Belgium; d School of Chemistry, University of East Anglia, Norwich Research Park, Norwich NR4 7TJ, UK; e School of Chemistry, University of St Andrews, KY16 9ST, UK; f School of Pharmacy, University of East Anglia, Norwich Research Park, Norwich NR4 7TJ, UK

**Keywords:** CPMV, cowpea mosaic virus, CYP, cytochrome P450, ELISA, Enzyme-Linked ImmunoSorbent Assay, EpHβA, 12,13β-epoxy,16β-hydroxy-β-amyrin, FPP, farnesyl diphosphate, FPS, farnesyl diphosphate synthase, GFP, green fluorescent protein, HMGR, 3-hydroxy,3-methylglutaryl-CoA reductase, HT, hyper-translatable, LPS, lipopolysaccharide, Nrf2, nuclear factor erythroid 2 related factor 2, SAD1, *Avena strigosa* β-amyrin synthase, SQE, squalene epoxidase, SQS, squalene synthase, tHMGR, truncated HMGR, TIC, total ion chromatogram, TNFα, tumor necrosis factor alpha, Transient plant expression technology, Synthetic biology, Terpenes, Triterpenoids, Combinatorial biosynthesis, Drug discovery

## Abstract

Plants are an excellent source of drug leads. However availability is limited by access to source species, low abundance and recalcitrance to chemical synthesis. Although plant genomics is yielding a wealth of genes for natural product biosynthesis, the translation of this genetic information into small molecules for evaluation as drug leads represents a major bottleneck. For example, the yeast platform for artemisinic acid production is estimated to have taken >150 person years to develop. Here we demonstrate the power of plant transient transfection technology for rapid, scalable biosynthesis and isolation of triterpenes, one of the largest and most structurally diverse families of plant natural products. Using pathway engineering and improved agro-infiltration methodology we are able to generate gram-scale quantities of purified triterpene in just a few weeks. In contrast to heterologous expression in microbes, this system does not depend on re-engineering of the host. We next exploit agro-infection for quick and easy combinatorial biosynthesis without the need for generation of multi-gene constructs, so affording an easy entrée to suites of molecules, some new-to-nature, that are recalcitrant to chemical synthesis. We use this platform to purify a suite of bespoke triterpene analogs and demonstrate differences in anti-proliferative and anti-inflammatory activity in bioassays, providing proof of concept of this system for accessing and evaluating medicinally important bioactives. Together with new genome mining algorithms for plant pathway discovery and advances in plant synthetic biology, this advance provides new routes to synthesize and access previously inaccessible natural products and analogs and has the potential to reinvigorate drug discovery pipelines.

## Highlights

• Transient plant expression technology provides rapid access to diverse triterpenes.
• Gram-scale quantities of purified triterpene can be generated.
• Agro-infiltration can be exploited for quick and easy combinatorial biosynthesis.
• Bespoke known and new-to-nature compounds can be generated.
• The platform allows isolation of analogs for structure-activity investigations.

## 1 Introduction

The Plant Kingdom harbors an enormous reservoir of diverse chemicals. Accessing these molecules promises to reinvigorate drug discovery pipelines and provide novel routes to synthesize complex compounds that are beyond the reach of synthetic chemistry Anarat-Cappillino and Sattely, 2014). Although breakthroughs in DNA sequencing technology and bioinformatics have accelerated discovery of candidate genes for new natural product pathways, effective harnessing of plant metabolic diversity will require: i) a quick and easy method to rapidly screen combinations of enzyme-encoding genes for production of novel structures by expression in a suitable heterologous host, and ii) the capability to purify suites of structural variants of these compounds in sufficient quantities for evaluation in bioactivity assays.

Microorganisms, particularly yeasts, have proven to be useful heterologous hosts for expression of several plant natural product pathways, including the artemisinin precursor artemisinic acid (Paddon et al., 2013), opioids (DeLoache et al., 2015, Galanie et al., 2015, Li and Smolke, 2016) and monoterpene indole alkaloids (Brown et al., 2015, Qu et al., 2015), and can be used for industrial-scale production of high-value plant products. However, despite some successes, many metabolites have only been produced at low levels in yeasts and optimization is far from trivial. For example, the development of a yeast platform for the production of artemisinic acid is estimated to have taken more than 150 person years (Kwok, 2010). Making microbes into efficient factories for the production of heterologous metabolites is hindered by lack of fundamental knowledge about cellular processes (Nielsen and Keasling, 2016).

Plants have a number of advantages as heterologous hosts for metabolic engineering. They require only simple inorganic nutrients, water, carbon dioxide and sunlight for efficient growth. They are also more amenable to expression of genes of plant origin than microbes since they support correct mRNA and protein processing, protein localisation and metabolic compartmentalisation, and already have many of the necessary metabolic precursors and co-enzymes. Species of tobacco are relatively easy and fast to transform by the integration of new genes into the genome but high yields can also be achieved in just a few days through transient expression following infiltration into the leaves of a culture of *Agrobacterium tumefaciens* carrying genes of interest on a binary plasmid vector, a process commonly known as agro-infiltration. *Nicotiana benthamiana*, a wild relative of tobacco, is particularly amenable to agro-infiltration-mediated transient expression and is currently being used for commercial production of flu vaccines (Marsian and Lomonossoff, 2016). The expression process is rapid, taking only 5–6 days from infiltration of leaves with *A. tumefaciens* strains harboring expression constructs to extraction of plant tissue. Importantly, combinations of genes can be co-expressed without the need for making multi-gene vectors simply by co-infiltrating multiple *A. tumefaciens* strains containing different expression constructs. Although various plant natural product pathways have previously been engineered by transient expression in this plant (Andersen-Ranberg et al., 2016, Geisler et al., 2013, Geu-Flores et al., 2009, Khakimov et al., 2015, Miettinen et al., 2014, Polturak et al., 2016, Rajniak et al., 2015, Wang et al., 2016), the levels achieved are usually in the microgram range. Here, we have optimized the *N. benthamiana* platform for production of up to gram-scale amounts of purified triterpene, and have demonstrated the power of this transient expression system for rapid and facile combinatorial biosynthesis by generating over 40 different types of oxygenated triterpene analogs. We further show the potential of this strategy for investigating structure-activity relationships in bioassays. This overall strategy will greatly accelerate systematic investigation of the vast array of triterpene diversity found in the Plant Kingdom by enabling the outputs of plant genomics to be translated into purified compounds for evaluation as drug leads and for other applications.

## 2 Results and discussion

### 2.1 Pathway engineering for increased triterpene yield in *N. benthamiana*

**Fig. 1:**
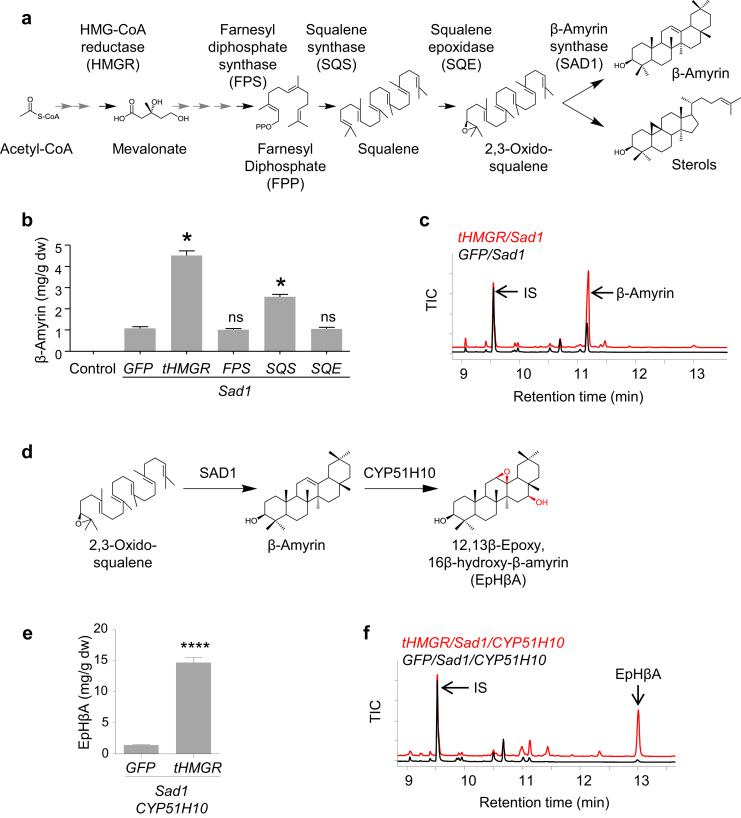
Co-expression with tHMGR gives enhanced triterpene levels **a**, Biosynthesis of triterpenes occurs via the mevalonate pathway. **b,** β-Amyrin content of tobacco leaves co-expressing SAD1 β-amyrin synthase with GFP, tHMGR, FPS, SQS or SQE (mean, three biological replicates ± s.e; control, GFP only); *, *P* < 0.05; n.s., not significant. **c**, Total ion chromatograms (TICs) for extracts from leaves expressing SAD1 with either GFP or tHMGR. **d**, Oxygenation of the β-amyrin scaffold to 12,13-epoxy, 16-hydroxy-β-amyrin (EpHβA) by CYP51H10. **e**, EpHβA content of leaves expressing SAD1 and CYP51H10 with GFP or tHMGR (mean, three biological replicates ± s.e.); ***, *P* < 0.0001. **f**, Total ion chromatograms (TICs) for the data shown in **e**. IS, internal standard (coprostan-3-ol).

The triterpenes are one of the largest classes of plant natural products (>20,000 reported to date) and are of considerable interest as potential drug leads (Hill and Connolly, 2012, Moses et al., 2014a). However few hypotheses exist regarding links between structure and activity because of the lack of available suites of structural analogs for bioactivity evaluation. These compounds are synthesized from the mevalonate pathway by cyclisation of the isoprenoid precursor 2,3-oxidosqualene to diverse triterpene scaffolds, the most common of which is β-amyrin (Thimmappa et al., 2014) (Fig. 1a). These scaffolds are then further modified by cytochromes P450 (CYPs) and other tailoring enzymes. Although yeast has been used as a heterologous expression host to make and modify triterpene scaffolds (Fukushima et al., 2013, Moses et al., 2014b, Thimmappa et al., 2014) the potential of *in planta* production has not been fully explored. We previously showed that transient expression of the oat β-amyrin synthase SAD1 in *N. benthamiana* leads to accumulation of β-amyrin in infiltrated leaf tissue (Geisler et al., 2013). Since precursor availability may be limiting, we co-expressed SAD1 with different upstream mevalonate pathway genes to determine the effects on triterpene production. Farnesyl diphosphate synthase (FPS) and squalene epoxidase (SQE) had little or no effect on β-amyrin content, while squalene synthase (SQS) gave a modest increase (Fig. 1b). The largest effect was seen with a feedback-insensitive version of HMG-CoA reductase (tHMGR), which gave a four-fold increase (Fig. 1b, c; Fig S1). CYP51H10 oxygenates β-amyrin at two positions to give 12,13-epoxy, 16-hydroxy-β-amyrin (EpHβA) (Fig. 1d) (Geisler et al., 2013). We used this CYP to evaluate the effects of co-expression with tHMGR on production of oxidized triterpene scaffolds by expressing SAD1 and CYP51H10 in *N. benthamiana* leaves with or without tHMGR, and showed that inclusion of tHMGR led to an increase in EpHβA production of ~10-fold (Fig. 1e,f; Fig S1). Thus, co-expression with tHMGR proved sufficient to significantly increase triterpene production in this transient plant expression system.

### 2.2 Gram-scale triterpene production using vacuum infiltration

**Fig. 2:**
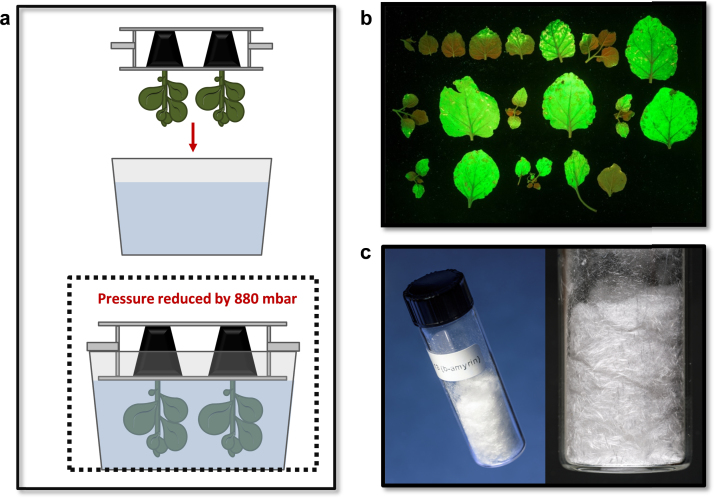
Generation of gram quantities of triterpene using vacuum infiltration **a**, Vacuum infiltration of *N. benthamiana* plants. Plants are retained by a bespoke holder, inverted into a bath containing 10 L of *A. tumefaciens* suspension, and a vacuum applied. Upon release of the vacuum the infiltration process is complete. **b**, GFP expression in leaves from a vacuum-infiltrated plant 5 days after infiltration (leaves arranged from top left to bottom right in descending order of their height on the plant). The youngest leaves (top left) were formed post-infiltration. **c**, β-Amyrin purified from vacuum-infiltrated plants following transient expression.

We next used *tHMGR* in combination with *Sad1* to investigate the potential of the transient plant expression platform for preparative-scale triterpene production. Our experiments up to this point had relied on infiltration of *N. benthamiana* leaves with *A. tumefaciens* cells using a syringe without a needle. To increase our capacity for effective leaf infiltration we designed a device that would enable efficient vacuum infiltration of 4–6 plants simultaneously (Fig. 2a). Preliminary experiments using a reporter construct showed that this method resulted in very good GFP expression in infiltrated leaves (Fig. 2b). We then carried out batch-wise infiltration of ~460 *N. benthamiana* plants with a mixture of two *A. tumefaciens* strains carrying CPMV-*HT* expression constructs for the *tHMGR* and *Sad1* genes, respectively, and purified >800 mg of β-amyrin as white needles (Fig. 2c). The crystals were estimated to have a purity of >98% by HPLC-CAD (Fig S2a). Given that the mother liquor still contained >150 mg of β-amyrin (Fig S2b), we estimate the total amount of β-amyrin recovered to be in the region of one gram, which corresponds to 0.4% dry leaf weight. These results demonstrate the potential of the *N. benthamiana* transient expression system for preparative-scale biosynthesis, with the opportunity for further scale-up, for instance by using larger vacuum infiltration devices.

### 2.3 Combinatorial biosynthesis using agro-infiltration provides rapid access to novel molecular diversity

Selective functionalization of triterpene scaffolds using synthetic chemistry is limited by lack of available reactive groups. Within the last few years considerable advances have been made in identifying and characterizing triterpene biosynthetic enzymes from plants (Biazzi et al., 2015, Boutanaev et al., 2015, Carelli et al., 2011, Fukushima et al., 2011, Fukushima et al., 2013, Geisler et al., 2013, Itkin et al., 2016, Khakimov et al., 2015, Miettinen et al., 2017, Moses et al., 2014b, Moses et al., 2014c, Moses et al., 2015a, Moses et al., 2015b, Salmon et al., 2016, Seki et al., 2008, Seki et al., 2015, Shang et al., 2014, Shibuya et al., 2006, Thimmappa et al., 2014, Yasumoto et al., 2016, Zhang et al., 2016), opening up opportunities to harness enzymes from nature for systematic investigation of a much broader sector of biologically relevant chemical space than can be achieved using conventional chemistry.

**Fig. 3:**
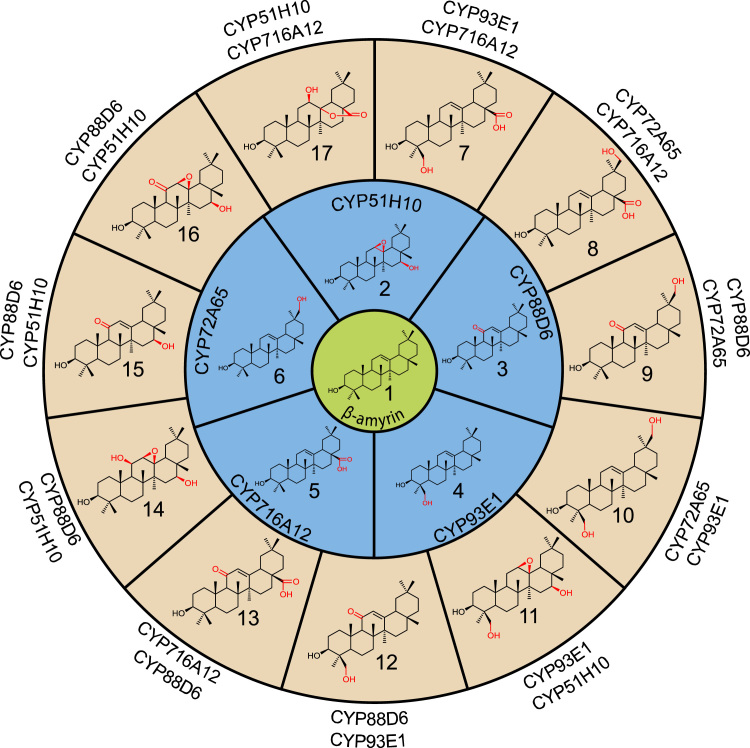
Combinatorial biosynthesis of oxygenated triterpenes in *N. benthamiana.* The major products of combinatorial biosynthesis are shown in the outer circle. The structures of compounds 11–17 were determined by NMR following initial GC-MS analysis (Figs S8-13; Tables S1-8). Structures 7–10 were inferred by GC-MS, based on comparison with previously reported compounds (Table S1; Figs S4-7). GC-MS also revealed a further 30 new unidentified products across the different enzyme combinations (Table S1). Similar results were obtained when β-amyrin synthase was co-expressed with the different CYP combinations in yeast (Fig. S14).

Since agro-infiltration enables co-expression of genes of interest without the need to make multi-gene constructs through co-infiltration of *A. tumefaciens* strains harboring different expression constructs, we evaluated the potential of the transient *N. benthamiana* expression system for combinatorial biosynthesis. We selected five CYPs from diverse plant species (oat, licorice, soy bean, barrelclover) that had previously been shown to oxidase β-amyrin at different scaffold positions (Carelli et al., 2011, Fukushima et al., 2011, Geisler et al., 2013, Moses et al., 2014c, Seki et al., 2008, Shibuya et al., 2006) and co-expressed each of these with tHMGR and SAD1 in *N. benthamiana*. All were functional and most converted the majority of available β-amyrin to the expected products (Fig. 3, blue circle; Fig S3). We then evaluated all ten possible pairwise combinations of these CYPs for further scaffold modification. A total of 41 products were detected across the different combinations (Figs S4-13; Table S1), many of which represented previously unreported structures. The major products generated are shown in Fig. 3. Compounds 7–10 were as predicted based on the known activities of the enzymes and matched the respective electron ionization spectra of previously reported triterpenes (Table S1; Figs S4-7). Compounds 11–17 were purified in quantities of 3.5–46 mg (Table S2) and their structures determined by NMR following initial GC-MS analysis (Figs S8-12; Tables S3-8). Two of these structures had previously been reported, whilst to our knowledge the other five had not (Table S1). These novel triterpenes included a C12β-hydroxyl, C13-C28-γ-lactone generated by co-expressing CYP51H10 with CYP716A12 (compound 17; Fig. 3), a product that has presumably arisen through intramolecular reactivity of the introduced functional groups. These examples illustrate the power of this highly versatile approach for rapid combinatorial expression and the potential for generating new molecules using biosynthetic enzymes from diverse plant species.

### 2.4 Preparative access to triterpene analogs allows investigation of structure-activity relationships

**Fig. 4:**
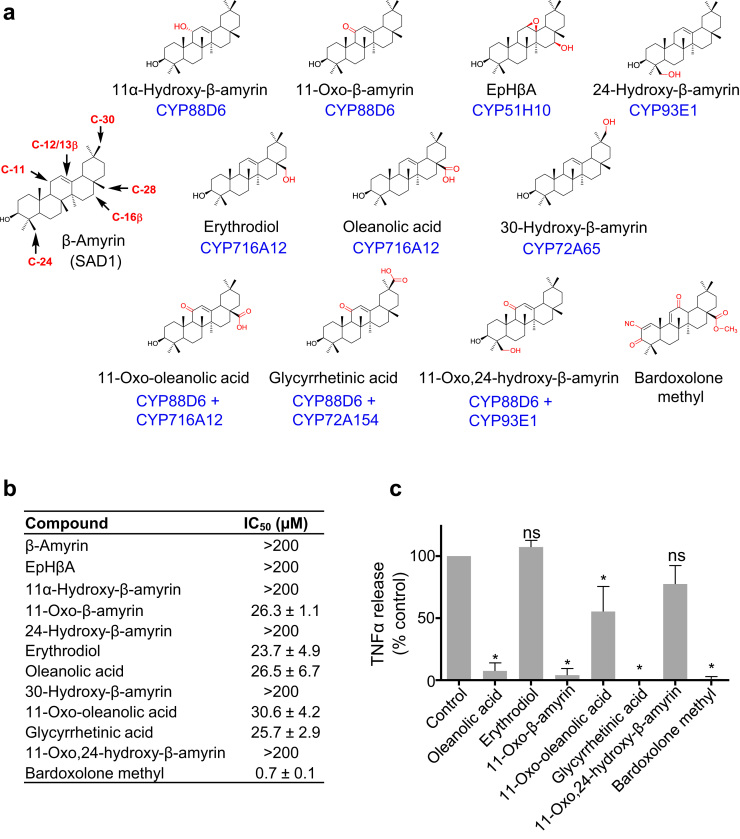
Evaluation of biological activity. **a**, Compounds used. **b**, Anti-proliferative assays. IC_50_ values (µM) for the human cancer cell line HL60 are shown (means, three biological replicates ± s.d.). **c**, Anti-inflammatory activity. TNFα release was measured by ELISA following lipopolysaccharide stimulation of THP-1 cells. Triterpenes were used at 100 μM, with the exception of bardoxolone methyl (100 nM). Values are relative to control DMSO-treated cells (means, three biological replicates ± s.d.). *, *P* < 0.05; n.s., not significant.

Oleanolic acid, a naturally occurring oxidized form of β-amyrin, has been used as a starting point to generate triterpene drug leads such as bardoxolone methyl (a potent activator of the Nrf2 pathway, currently in phase 2 clinical trials) using synthetic chemistry (Liby and Sporn, 2012). However, these initial endeavors were constrained by lack of oxidative functionality on the scaffold. Using our toolkit of triterpene biosynthetic enzymes in combination with plant transient expression technology we have greatly expanded the possibility for creating diversified suites of oxidized variants in sufficient quantities for bioassays. As a proof of concept, we assayed a series of oxygenated β-amyrin analogs that we had purified from our *in planta* expression system along with several commercially available triterpenes (oleanolic acid, erythrodiol, glycyrrhetinic acid) and demonstrated differences in anti-proliferative and anti-inflammatory activity using human cell lines (Fig. 4). Although the activity of these molecules was orders of magnitude lower than that of bardoxolone methyl, these experiments demonstrate that small differences in the type and position of oxidation can result in marked differences in bioactivity and open up opportunities to further diversify the oleanane scaffold (by further metabolic engineering and/or synthetic chemistry), so enabling systematic investigation of structure-activity relationships for pharmaceutical applications.

## 3 Conclusions

There are more than 400,000 species of flowering plants in the world. The vast majority of these (>99.9%) have not yet been sequenced. Over 50% of drugs in current use are derived from natural products (Harvey et al., 2015). As methods for sequencing and mining plant genomes are becoming faster, cheaper and more effective, candidate biosynthetic pathways for multitudes of new natural products will undoubtedly be revealed (Medema and Osbourn, 2016, Owen et al., 2017).

Plants have many inherent advantages over microbes for expression of genes, enzymes and pathways of plant origin. However, they have historically been overlooked as efficient heterologous hosts for small molecule production, in part due to the technical and regulatory challenges and length of time associated with generation of high-level producing stable transgenic lines. Although *N. benthamiana* has previously been used as a host for transient expression of terpene biosynthetic genes (primarily for mono-, di- and sesquiterpenes), the estimated levels as assessed by GC- or LC-based quantification of leaf extracts were in the region of 0.6 μg – 1 mg/g dry weight. Most of these are theoretical yields since few of these products were purified, which would inevitably result in significant yield loss (Table S11). In a proof of concept experiment we previously showed that we could purify 20 mg of triterpene from 17 g of leaf tissue following transient expression in *N. benthamiana* (an actual yield of 1.18 mg/g dry weight) (Geisler et al., 2013). Here we demonstrate optimization of this expression system through a combination of pathway engineering and improvement of the agro-infiltration process. We firstly show that inclusion of a feedback insensitive form of HMG-CoA reductase in our co-infiltration experiments increased the yields of simple and oxidized triterpenes by 4–10 fold. Secondly, we develop a highly effective vacuum infiltration process and demonstrate that this can be used for infiltration of whole plants rather than individual leaves, to yield gram-scale quantities of purified triterpene. Thirdly, we show that this transient expression system can be used to rapidly generate suites of purified triterpenes and analogs, simply by mixing *A. tumefaciens* strains carrying different expression constructs.

Our work demonstrates the power and potential of this novel plant transient transfection technology for rapid preparative access to plant-derived molecules. This technology, in combination with advances in commercial DNA synthesis and establishment of standardized methods and pipelines for modular and rapid construction of vectors for heterologous expression in plants using synthetic biology approaches (Patron et al., 2015, Owen et al., 2017), opens up unprecedented opportunities for harnessing new molecular diversity for drug discovery, producing normally low-abundance molecular entities in quantity (enablement of bioprospecting hits), and improving on promising natural product leads (lead optimization for complex biomolecules).

## 4 Materials and methods

### 4.1 Generation of Gateway Entry constructs

Oligonucleotide primers were purchased from Sigma-Aldrich and all PCR steps were performed using Phusion polymerase (New England Biolabs). The oat *HMGR, FPS, SQS* and *SQE* gene sequences were identified from an *A. strigosa* root tip 454 transcriptomic database (Kemen et al., 2014) (GenBank accession numbers *tHMGR* – KY284573; *FPS1 –* KY284574; *SQS1* – KY284575; *SQE1* – KY284576). *A. strigosa* root tip RNA extraction and cDNA synthesis was performed as described previously (Kemen et al., 2014). The partial *HMGR* gene lacking the first 417 nucleotides (*tHMGR*) was amplified from this cDNA using primers P1 + P2 (Table S10) and cloned into the Gateway Entry vector pENTR-D-TOPO (Invitrogen) according to the manufacturer's instructions. Full length coding sequences of *FPS, SQS* and *SQE* were amplified from cDNA by PCR using primers P3 + P4, P5 + P6 and P7 + P8 respectively (Table S10). The amplified products were inserted into a Zero Blunt TOPO vector (Invitrogen) according to the manufacturer's instructions. These vectors were then used as templates for PCR to generate Gateway-compatible inserts using primers P9 + P10, P11 + P12 and P13 + P14 respectively (Table S10). The amplified products were cloned into the pDONR207 vector using BP clonase II enzyme mix (Invitrogen) according to the manufacturer's instructions. SAD1 and CYP51H10 Gateway inserts were likewise amplified from vectors as described previously (Geisler et al., 2013) using the primers P15 + P16 and P17 + P18 respectively and inserted into pDONR207 (Table S10). The full length coding sequence for *CYP72A65* (Medtr8g042000) was amplified from *M. truncatula* cDNA using primers P19 and P20 and then inserted into pDONR221 (Thermo Fisher). All constructs were sequenced within the Entry vector to verify the integrity of the clones (performed by Eurofins Genomics). The *CYP88D6* entry clone (Seki et al., 2008) was kindly donated by Prof. Toshiya Muranaka. Entry clones for *CYP716A12* (Moses et al., 2014b) and *CYP93E1* (Moses et al., 2014c) were used as previously described.

Genes were cloned from the relevant Entry vectors into pEAQ-*HT*-DEST1 using LR clonase II enzyme (Invitrogen) according to the manufacturer's instructions. An expression construct containing GFP in pEAQ-HT-DEST1 (Sainsbury et al., 2009) was kindly provided by the Lomonossoff laboratory (John Innes Centre, UK). The expression constructs were transformed into chemically competent *Agrobacterium tumefaciens* strain LBA4404 by flash freezing in liquid nitrogen.

### 4.2 Agro-infiltration

A. tumefaciens strains carrying the relevant constructs were cultured and prepared for infiltration as described previously (Sainsbury et al., 2012) except that the OD_600_ of the cells in MMA buffer was adjusted to 0.2 x **n**, depending on the number of cultures (**n**) to be coinfiltrated. Bacterial suspensions were always combined in equal volumes for coinfiltration. N. benthamiana plants were grown as in Sainsbury et al. (2012). Infiltration by syringe was performed as previously described (Sainsbury et al., 2012). For vacuum infiltration, the infiltration apparatus was constructed from a vacuum oven (LTE Scientific) which formed the infiltration chamber. The oven was connected to a pump via a vacuum reservoir (AVE) to reduce the time required to achieve the desired vacuum in the infiltration chamber. Plants were secured within a bespoke holder and inverted into a stainless steel water bath containing 10 L of A. tumefaciens suspension, ensuring that the aerial parts were completely submerged. The water bath was sealed within the vacuum chamber, and the vacuum applied for approximately one minute until the pressure had been reduced by 880 mbar. The infiltration chamber was then quickly returned back to atmospheric pressure resulting in infiltration of the bacterial suspension. Infiltrated leaves were harvested after 5 days.

### 4.3 Quantification of β-amyrin and EpHβA in *N. benthamiana* leaf extracts

Infiltrated *N. benthamiana* leaves were harvested and 12 mm leaf disks were punched and frozen at −80 °C. The disks were lyophilized, weighed and transferred to 2 mL GC-MS autosampler vials (Agilent). Standard solutions of β-amyrin (Extrasynthese) and EpHβA (this study) were made to a concentration of 0.5 mg/mL in EtOH and used to make ten 2-fold dilutions, which when dried gave a calibration series from 50 μg down to ~0.1 μg. These steps were performed using a Gerstel MultiPurpose Sampler (MPS) to minimize pipetting errors. A saponification solution (EtOH:H_2_O:KOH 9:1:1 v: v:w) was prepared containing the internal standard (coprostan-3-ol, Sigma-Aldrich) to a final concentration of 10 μg/mL. Aliquots (500 μL) were pipetted into each sample vial (leaf disks and standards) using the Gerstel MPS. Samples were incubated at 65 °C for 2 h with intermittent agitation. 250 μL of H_2_O was added to each sample and vortexed before addition of 500 μL hexane. Samples were vortexed (2 × 10 s) and 100 μL of the upper hexane phase transferred to a fresh vial and dried. GC-MS analysis was performed as described in section 3.5 and data analyzed using Agilent MassHunter Quantification software. Quantification of β-amyrin and EpHβA was achieved relative to the external standard calibration curves. Quantifier ions for coprostan-3-ol (370), β-amyrin (218) and EpHβA (189) were used. Quantification values were divided by the weight of the original leaf disk to calculate yield per gram of dry leaf weight. Graphs were constructed using GraphPad Prism (version 5). Statistics were performed using one-way ANOVA with post-hoc analysis (Tukey's multiple comparisons test).

### 4.4 Combinatorial triterpene biosynthesis

Five-week-old plants were infiltrated using three leaves per plant as replicates. For each combination, four *A. tumefaciens* strains were coinfiltrated including two carrying *tHMGR* and *Sad1* along with two strains carrying the relevant CYP genes (where a single CYP was used, GFP was included in place of the second CYP). Leaves were harvested 5 days after infiltration and lyophilized. For extraction, a single 15 mm leaf disk was taken from each dried leaf and 500 μL EtOAc [containing 10 μg/mL coprostan-3-ol (Sigma-Aldrich)] added to each sample. Leaf tissue was disrupted by shaking with a single 3 mm tungsten carbide bead (Qiagen). Samples were incubated at room temperature with gentle shaking for 2 h. Aliquots (50 μL) were taken from each sample, transferred to a separate vial and dried under N_2_. GC-MS analysis was performed as described in section 3.5 and samples analyzed using Agilent MassHunter Qualitative Analysis software. New products were identified by overlaying the total ion chromatograms of the combinatorial samples with their relevant single CYP controls (see Fig. S4-S13). A summary of the combinatorial biosynthesis results is provided in Table S1.

### 4.5 GC-MS analysis of leaf extracts

Dried samples were derivatized using Tri-Sil Z reagent (Sigma-Aldrich) and diluted in an equivalent volume of ethyl acetate prior to GC-MS analysis. GC was performed using an Agilent 7890B fitted with a Zebron ZB5-HT Inferno column (Phenomenex). Injections were performed in pulsed splitless mode (30 psi pulse pressure) with the inlet temperature set to 250 °C. The GC oven temperature program was 170 °C and held for 2 mins with subsequent increase to 300 °C (20 °C/min) and held at 300 °C for an additional 11.5 min (total run time 20 min). The GC oven was coupled to an Agilent 5977 A Mass Selective Detector set to scan mode from 60 to 800 mass units (solvent delay 8 min). For quantification, the detector was set to 7.2 scans/sec.

### 4.6 Triterpene purification

Infiltrations were performed as described in section 3.2 using six-week-old *N. benthamiana* plants. A summary of infiltrated *A. tumefaciens* strains, plant quantities and product yields is provided in Table S2. Details of purification of specific products are provided in Supplementary Information.

### 4.7 NMR analysis

NMR spectra were recorded in Fourier transform mode at a nominal frequency of 400 MHz for ^1^H NMR and 100 MHz for ^13^C NMR, using the specified deuterated solvent. Chemical shifts were recorded in ppm and referenced to the residual solvent peak or to an internal TMS standard. Multiplicities are described as, s = singlet, d = doublet, dd = doublet of doublets, dt = doublet of triplets, t = triplet, q = quartet, m = multiplet, br = broad, appt = apparent; coupling constants are reported in hertz as observed and not corrected for second order effects. NMR data are provided as supplementary information.

### 4.8 Yeast strains and metabolite analysis

Yeast strains for combinatorial biosynthesis were generated from strain PA14 (Miettinen et al., 2017). Strain 1 (Table S9) was generated by transforming PA14 with a yeast expression construct harboring the *Glycyrrhiza glabra* β-amyrin synthase GgbAS, the *M. truncatula* cytochrome P450 reductase (MTR1) and the truncated *S. cerevisiae* HMGR1, pESC-URA[GAL1/GgbAS T2A MTR1; GAL10/tHMG1] (Miettinen et al., 2017). For generating CYP yeast expression vectors, the Gateway Entry vectors carrying *CYP51H10, CYP88D6, CYP72A65* and *CYP716A12* as described in section 3.1 were used. A vector carrying the functionally equivalent *CYP93E9* (Moses et al., 2014c) was used in place of *CYP93E1*. These were recombined into both yeast expression destination vectors pAG423GAL-ccdB (Addgene plasmid 14149 (Alberti et al., 2007)) and pAG425GAL-ccdB (Addgene plasmid 14153 (Alberti et al., 2007)). These vectors were transformed into PA14 and the yeast strains cultivated in the presence of methyl-β-cyclodextrins (MβCD) as previously described (Moses et al., 2014b). Aliquots (10 mL) of spent yeast culture medium were extracted twice with 5 mL hexane and then with 5 mL of ethyl acetate. The resulting organic extracts were pooled and evaporated to dryness. Samples were derivatized with 50 μL of N-methyl-N-(trimethylsilyl) trifluoroacetamide (Sigma-Aldrich) and 10 μL of pyridine. GC-MS analysis was carried out as previously described (Moses et al., 2014b).

### 4.9 Bioactivity assays

β-Amyrin, oleanolic acid, erythrodiol and 18β-glycyrrhetinic acid were purchased as dry powders from Extrasynthese. Bardoxolone-methyl was purchased as a dry powder from SelleckChem. Products purified from *N. benthamiana* were dried under vacuum for 24 h to ensure removal of trace solvents. Stock solutions of 10 or 20 mM were made in DMSO under sterile conditions. Eight two-fold serial dilutions of these stocks were then made (in DMSO) for use in bioassays. Sterile conditions were maintained throughout all subsequent experiments. HL60 and THP-1 cell lines were obtained from the European Collection of Authenticated Cell Cultures (ECACC). These were maintained in Roswell Park Memorial Institute (RPMI) 1640 media (Hyclone) supplemented with 10% heat inactivated bovine fetal calf serum (Hyclone), l-glutamine (2 mM) and antibiotics (penicillin 100 U/mL; streptomycin (100 μg/mL) (Gibco). Cultures were maintained at 37 °C in a humidified atmosphere with 5% CO_2_ and passaged every 3.5 days. Cell densities were measured using a Malassez hemocytometer under a light microscope and diluted by addition of fresh media to the densities described below.

For anti-proliferation assays, HL60 cells were seeded at a density of 3x10^5^ cells/mL in 100 μL aliquots in 96 well plates (3x10^4^ cells per well). To each 100 μL well, 1 μL of the test compound was added from the series of stock solutions described above to give the final test concentration (in the micromolar range). Each plate included controls of untreated cells, vehicle (DMSO)-treated controls and media only-wells. Following addition of the compounds, the cells were incubated for 72 h at 37 °C (with 5% CO_2_). Cell proliferation was determined using a Cell Titer 96 Aqueous non-radioactive assay; (Promega). MTS reagent was added to the wells and incubated for 4 h before measuring absorbance at 492 nm. Absorbance values were corrected by subtracting the media-only control. IC50 values were calculated using non-linear regression analysis performed in GraphPad Prism software (version 5).

For the anti-inflammatory assays, THP-1 cells were seeded at a density of 1x10^6^ cells/mL in 500 μL aliquots (5x10^5^ cells per well). Test compounds (100 μM aliquots) were added to the appropriate wells and the cells incubated for 30 min at 37 °C (5% CO_2_). After this time, 1 μL of LPS (Merck Millipore) was added (from a 0.5 mg/mL stock solution in RPMI medium) to the appropriate wells to stimulate TNFα production. The cells were incubated for a further 3 h before pelleting by centrifugation (2000 rpm, 5 min). Supernatants were harvested, transferred to fresh tubes on ice, and stored at −80 °C until needed. The TNFα ELISA was performed using a Human TNF ELISA kit (BD Biosciences) according to the manufacturer's instructions. Aliquots (100 μL) of the supernatants (diluted 4-fold) and standards were added to the ELISA plate. A TNFα standard (BD Biosciences) was prepared across a two-fold serial dilution range from 500 pg/mL to 7.8 pg/mL (diluted in phosphate buffered saline solution: per liter: 8 g NaCl, 1.17 g Na_2_HPO_4_, 0.2 g KH_2_PO_4_, 0.2 g KCl) and used to generate a standard curve for determining TNFα production. Plates were incubated at room temperature for 2 h before washing and detection of TNFα with antibodies using the TMB substrate reagent kit (BD Biosciences) according to the manufacturer's instructions. The absorbance of the samples was measured at 450 nm with subtraction of absorption values at 570 nm. Quantification of TNFα in each sample was performed by linear regression analysis of the standard curve using GraphPad Prism (version 5). Statistical analysis was performed using a one-way ANOVA test with post-hoc analysis (Tukey's multiple comparisons test).

## Author contributions

J.R. carried out the optimization of the *N. benthamiana* platform for triterpene production, *in planta* combinatorial biosynthesis, triterpene extraction and analysis and the bioassay work; M.J.S. performed chemical purification and NMR; K.M. carried out yeast expression, combinatorial biosynthesis and triterpene analysis; B.B. established the vacuum infiltration method for *N. benthamiana*; A.L. designed and made expression constructs; P.B. advised and assisted with GC-MS analysis; R.J.M.G. contributed to the design of the project; A.G. contributed to design and analysis of the yeast expression experiments; M.A.O’C. contributed to the design and analysis of the bioassays; A.O. contributed to experimental design and data analysis. All authors contributed to the preparation of the manuscript.

## Author information

The authors declare no competing financial interests. Correspondence and requests for materials should be addressed to A.O. (anne.osbourn@jic.ac.uk).
