# Supplementary material for: A translational synthetic biology platform for rapid access to gram-scale quantities of novel drug-like molecules
Source: Metab Eng. 2017 Jul;42:185–93. doi: 10.1016/j.ymben.2017.06.012 (PMC5555447; doi:10.1016/j.ymben.2017.06.012)
Supplement: Supplementary file 1 — Supplementary material [file mmc1.docx]

**Supplementary Information for:**

A translational synthetic biology platform for rapid access to gram-scale quantities of novel drug-like molecules

James Reed, Michael J. Stephenson, Karel Miettinen, Bastiaan Brouwer, Aymeric Leveau, Paul Brett, Rebecca J.M. Goss, Alain Goossens, Maria A. O’Connell and Anne Osbourn.

**Contents:**

Supplementary methods

Supplementary Figures S1-S14

Supplementary Tables S1-S10

# Supplementary methods

*General considerations for compound extraction and purification*

All harvested leaf material was lyophilized and ground to a powder in liquid N_2_ before extraction. Solvents used for extraction and flash chromatography were reagent grade and used without further distillation or drying unless otherwise stated. HPLC mobile phases were prepared using HPLC grade solvents. Flash chromatography grade Silica gel 60 (Material Harvest) was used unless otherwise stated. Thin layer chromatography (TLC) was performed on aluminum plates coated with 0.2 mm silica gel-60 F_254_ (Sigma-Aldrich). TLC plates were visualized under UV light (254 nm) followed by staining with p-anisaldehyde (2 % v/v p-anisaldehyde, 2% v/v concentrated H_2_SO_4_, in acetic acid) or vanillin (6% w/v vanillin, 1.5 % v/v concentrated H_2_SO_4_ in 96 % aqueous EtOH) stain. All flash chromatography steps were performed using an Isolera One (Biotage).

For processing of leaf material by pressurized extraction, dried *N. benthamiana* powder was dispersed with 1 part (by volume) of quartz sand (0.3-0.9 mm), and this mixture was layered on top of a bottom layer of quartz sand (0.3-0.9 mm) 3 cm in depth within a 120 mL extraction cell. Extraction was performed using a SpeedExtractor E-914 (Büchi) with three cycles at 100 °C and 130 bar pressure. Cycle one had zero hold time, and cycles two and three had 5 min hold times. The run finished with a 2 min solvent flush and 6 min N_2_ flush.

For saponification of leaf material, the saponification solution (8:2 EtOH:50 % w/v aq. KOH solution) was added to the dry leaf material and heated at 65 °C for two hours. The EtOH was reduced by rotary evaporation before addition of water and extraction with EtOAc.

For Ambersep treatment, Ambersep 900 OH resin was purchased from Sigma-Aldrich and used as supplied. Crude extracts were dissolved in the minimum volume of EtOH possible, 50 mL of resin beads were added and the mixture was shaken at room temperature. Additional 50 mL aliquots of resin beads were added every 30 mins until the green coloration had been removed. The resin was removed by filtration through a short column of diatomaceous earth (Celite, Sigma-Aldrich). The resin beads and diatomaceous earth were rinsed sequentially with EtOH, EtOH:hexane, and hexane twice (The volume of each rinse being sufficient to wet the recovered resin bed). The filtrate and rinses were combined and concentrated to dryness by rotary evaporation under vacuum.

*Preparative production of β-amyrin*

Dry leaf powder (244 g) was extracted batch-wise with EtOH using pressurized solvent extraction and chlorophyll removed by Ambersep 900 OH treatment. After filtration and drying, the crude solid (38 g) was leached with 500 mL dichloromethane (DCM) for 2 h at room temperature with occasional agitation. The leachate was filtered through a short column (6 x 20 cm) of silica gel 60 (Material Harvest), eluted with DCM, and 250 mL fractions collected. Fractions containing β-amyrin (as assessed by TLC) were combined and concentrated to give 4.7 g of an orange amorphous solid. The crude solid was subjected to flash chromatography (silica gel column dimensions 5 x 11 cm, mobile phase DCM isocratic, 100 mL fractions), and fractions containing β-amyrin (as assessed by TLC) combined and concentrated to dryness, affording 2.55 g of a light orange amorphous solid. The solid was dissolved in 50 mL DCM, and 250 mg decolorizing charcoal (Sigma-Aldrich) was added. After 30 mins at room temperature with occasional agitation, the mixture was filtered through a short column (1 x 5 cm) of diatomaceous earth (Sigma-Aldrich) and dried, to give 2.4 g of light yellow amorphous solid. Finally the solid was crystallized from hot MeOH, yielding 809 mg of β-amyrin as white needles. The properties of this product were as follows: Mp 197.2-198.0 ^o^C (lit. 196–198°C). ^1^H NMR (CDCl_3_, 400 MHz) *δ* 5.18 (1H, t, *J* = 3.60), 3.26-3.18 (1H, brm), 2.04-1.19 (17H, m), 1.14 (3H, s), 1.12-0.71 (6H, m), 1.00 (3H, s), 0.97 (3H, s), 0.94 (3H, s), 0.87 (6H, s), 0.83 (3H, s), 0.79 (3H, s)*.* ^13^C NMR (CDCl_3_, 100 MHz) *δ* 145.2, 121.7, 79.0, 55.2, 47.6, 47.2, 46.8, 41.7, 39.8, 38.8, 38.6, 37.1, 37.0, 34.7, 33.3, 32.7, 32.5, 31.1, 28.4, 28.1, 27.3, 26.9, 26.2, 26.0, 23.7, 23.5, 18.4, 16.8, 15.6, 15.5 (identical to commercial standard (Extrasynthese)). The purified product was diluted to 0.5 mg/mL in tetrahydrofuran (THF) and analyzed by HPLC-CAD. The HPLC was fitted with a Phenomenex Kinetex 2.6 μm XB-C18 100 Å, 50 x 2.1 mm column at 40 ^o^C. CAD detector sampling was 5 Hz. An aliquot (1 µL) of the sample was injected using a mobile phase gradient as follows: Solvent A: [water] Solvent B: [MeOH]; gradient: 25% [B] to 100% [B] from 0 to 10 min, 100% [B] hold from 10 to 15 mins, 100% [B] to 25% [B] from 15 to 17 mins, 25% [B] hold from 17 to 22 mins; flow rate: 0.4 mL/per min. β-Amyrin was found to have a retention time of 11.18 min (Figure S2). The remaining mother liquor was contracted and subjected to flash chromatography [SNAP Ultra 50 g normal-phase column (Biotage), eluent DCM isocratic, 9 mL fractions]. Fractions containing β-amyrin (as assessed by TLC) were combined and concentrated by rotary evaporation under vacuum, affording 325 mg of an off-white oil. The oil was estimated to be comprised of 48% β-amyrin by HPLC-CAD (Figure S2).

*Isolation of EpHβA*

Dry leaf powder (Table S2) was saponified and the EtOAc extract dried by rotary evaporation and dissolved in a small volume of toluene. Flash chromatography was performed (10 g SnapUltra silica (Biotage) as follows: Solvent A: [Hexane] Solvent B: [EtOAc]; gradients: 0% [B] to 6% [B] over 2 column volumes, 6% [B] to 100% [B] over 10 column volumes, 100% [B] for 7 column volumes; flow rate: 36 mL/min, 14 mL fractions). Fractions were analyzed by GC-MS and TLC and those containing EpHβA pooled and adsorbed onto silica gel from DCM. Additional flash chromatography was performed [10 g SnapUltra silica (Biotage)]: Solvent A: [hexane] Solvent B: [EtOAc]; gradient: 0% [B] to 100% [B] over 60 column volumes; flow rate: 36 mL/min, 14 mL fractions). Again, fractions were analyzed by TLC and GC-MS and those with the highest abundance of EpHβA combined to give ~100 mg of a yellow solid. This crude solid was decolorized using activated charcoal (Sigma-Aldrich) before crystallization with hot EtOH to give a total of 79 mg of white crystals of EpHβA. This product had an identical retention time and mass spectra to the CYP51H10 product in *N. benthamiana* crude leaf extracts (Figure S1) and a previously reported mass spectrum (Geisler et al., 2013).

*Isolation of 11α-hydroxy-β-amyrin and 11-oxo-β-amyrin*

Dry leaf powder (Table S2) was saponified and the EtOAc extracts dried and dissolved in a small volume of toluene. Flash chromatography was performed (10 g SnapUltra silica (Biotage): Solvent A: [Hexane] Solvent B: [EtOAc]; gradient: 0% [B] to 6% [B] over 2 column volumes, 6% [B] to 50% [B] over 6 column volumes, 50% [B] to 100% [B] for 1 column volume, 100 % [B] for 5 column volumes; flow rate: 36 mL/min, 14 mL fractions). Fractions were analyzed by GC-MS to identify those containing 11α-hydroxy-β-amyrin and 11-oxo-β-amyrin and the respective fractions pooled. Further flash chromatography was performed on each of these pooled fractions (10 g SnapUltra silica (Biotage): Solvent A: [Hexane] Solvent B: [EtOAc]; gradient: 0% [B] to 7% [B] over 1 column volumes, 7% [B] to 70% [B] over 10 column volumes, 7% [B] for 2 column volumes; flow rate: 36 mL/min, 14 mL fractions). This was performed twice for each compound. These steps led to isolation of 7 mg of off-white crystals of 11α-hydroxy-β-amyrin and approximately 20 mg of a yellow solid for 11-oxo-β-amyrin. 11-Oxo-β-amyrin was further purified by crystallization using hot EtOH to give 10.4 mg of white crystals. The spectra of the isolated products were found to match those of published GC-MS spectra of 11α-hydroxy-β-amyrin and 11-oxo-β-amyrin (Seki et al., 2008).

*Isolation of 24-hydroxy-β-amyrin*

The dry leaf powder (Table S2) was saponified and EtOAc extracts were dried and dissolved in a small volume of toluene prior to flash chromatography [10 g SnapUltra silica (Biotage): Solvent A: [hexane] Solvent B: [EtOAc]; gradient: 0% [B] to 6% [B] over 2 column volumes, 6% [B] to 50% [B] over 6 column volumes, 50% [B] to 100% [B] for 1 column volume, 100% [B] for 5 column volumes; flow rate: 36 mL/min, 14 mL fractions]. Fractions containing impure 24-hydroxy-β-amyrin were pooled and dried before further flash chromatography (10 g Biotage SnapUltra silica: Solvent A: [hexane] Solvent B: [EtOAc]; gradient: 0% [B] to 7% [B] over 1 column volume, 7% [B] to 70% [B] over 10 column volumes, 7% [B] for 2 column volumes; flow rate: 36 mL/min, 14 mL fractions). This allowed isolation of fractions containing 24-hydroxy-β-amyrin which when dried gave 4.7mg of a white crystalline solid which matched published GC-MS spectra of 24-hydroxy-β-amyrin (Moses et al., 2014c).

*Isolation of 30-hydroxy-β-amyrin*

Dry leaf powder (Table S2) was saponified and the EtOAc extract was dried and dissolved in a small volume of toluene before flash chromatography [10 g SnapUltra silica (Biotage): Solvent A: [hexane] Solvent B: [EtOAc]; gradient: 0% [B] to 6% [B] over 2 column volumes, 6% [B] to 50% [B] over 6 column volumes, 50% [B] to 100% [B] for 1 column volume, 100% [B] for 5 column volumes; flow rate: 36 mL/min, 14 mL fractions). The fractions were monitored by GC-MS and TLC to identify those containing 30-hydroxy-β-amyrin which were pooled, dried and again dissolved in toluene for flash chromatography (10 g SnapUltra silica (Biotage): Solvent A: [hexane] Solvent B: [EtOAc]; gradient: 0% [B] to 10% [B] over 1 column volume, 10% [B] to 80% [B] over 10 column volumes, 8% [B] for 2 column volumes; flow rate: 36 mL/min, 14 mL fractions). Fractions containing 30-hydroxy-β-amyrin were again pooled and dried to give 8.9 mg of an orange solid. This solid was triturated in MeOH at room temperature to remove the orange impurities leaving 6.5 mg of a white solid. The isolated product matched published GC-MS spectra of 30-hydroxy-β-amyrin (Seki et al., 2011).

*Isolation and structural determination of 12,13β-epoxy,16β,24-dihydroxy-β-amyrin*

Dry leaf powder (Table S2) was extracted with EtOAc using pressurized solvent extraction and subjected to Ambersep 900 OH treatment before fractionation by flash chromatography (120 g SiliaSep cartridge (Silicyle) as follows: Solvent A: [Hexane] Solvent B: [EtOAc]; gradient: 0% [B] to 100% [B] over 3000 mL; flow rate: 100 mL/min, 250 mL fractions). The fraction containing 12,13β-epoxy,16β,24-dihydroxy-β-amyrin (identified by GC-MS) was concentrated and further fractionated by flash chromatography using 2x10 g SnapUltra silica cartridge (Biotage) stacked together as follows: Solvent A: [Hexane] Solvent B: [EtOAc]; gradient: 30% [B] to 70% [B] over 3080 mL; flow rate: 36 mL/min, 22 mL fractions). Fractions containing 12,13β-epoxy,16β,24-dihydroxy-β-amyrin (as assessed by GC-MS) were combined, concentrated and treated with activated charcoal. After filtration on a short column of diatomaceous earth, the solution was concentrated to dryness and triturated in cold MeOH, affording 17.0 mg of 12,13β-epoxy,16β,24-dihydroxy-β-amyrin as a white amorphous solid. The structure was determined as 12,13β-epoxy,16β,24-dihydroxy-β-amyrin by NMR. Assignments were made using a combination of ^1^H, ^13^C, DEPT-edited HSQC, HMBC and 2D NOESY experiments (Table S3).

*Isolation and structural determination of 11-oxo, 24-hydroxy-β-amyrin.*

Dry leaf powder (Table S2) was saponified and the EtOAc extract adsorbed onto silica gel prior to flash chromatography (25 g SNAP KP-Sil cartridge, Solvent A: [Hexane] Solvent B: [EtOAc]; gradient: 0 % [B] to 5 % [B] over 2 column volumes, 5% [B] to 100 % [B] over 18 column volumes, 100 [B] for 2 column volumes; flow rate: 75 mL/min, 14 mL fractions). Fractions containing 11-oxo, 24-hydroxy-β-amyrin (as assessed by GC-MS) were combined, concentrated and subjected to additional fractionation by flash chromatography (10 g SnapUltra silica (Biotage): Solvent A: [Hexane] Solvent B: [EtOAc]; gradient: 0 % [B] to 20 % [B] over 20 column volumes, 20% [B] to 100 % [B] over 3 column volumes, 100 [B] for 5 column volumes; flow rate: 36 mL/min, 14 mL fractions). Fractions containing 11-oxo, 24-hydroxy-β-amyrin (as assessed by GC-MS) were combined and concentrated to give 41.0 mg of product as a white amorphous solid. The structure was determined by NMR. Assignments were made via a combination of ^1^H, ^13^C, DEPT-edited HSQC, HMBC and 2D NOESY experiments (see Table S4).

*Isolation and structural determination of 11-oxo-oleanolic acid*

Dry leaf powder (Table S2) was saponified and the EtOAc extracts combined and evaporated to dryness. The crude solid was adsorbed onto silica gel (Material Harvest) and subjected to fractionation by flash chromatography (50 g SNAP Ultra cartridge: Solvent A: [Hexane] Solvent B: [EtOAc]; gradient: 0% [B] to 5% [B] over 2 column volumes, 5% [B] to 100% [B] over 15 column volumes, 100 [B] for 5 column volumes; flow rate: 75 mL/min, 14 mL fractions). Fractions containing 11-oxo-oleanolic acid (as assessed by GC-MS) were combined, concentrated, and subjected to additional fractionation by flash chromatography (10 g SnapUltra silica (Biotage): Solvent A: [Hexane] Solvent B: [EtOAc]; gradient: 0% [B] to 5% [B] over 1 column volumes, 5% [B] to 40% [B] over 15 column volumes, 40% [B] to 100% [B] for 2 column volumes, 100% [B] for 5 column volumes; flow rate: 36 mL/min, 14 mL fractions). Fractions containing 11-oxo-oleanolic acid (as assessed by GCMS) were combined, concentrated affording 46.0 mg of product as a white amorphous solid. The compound was analyzed by NMR and found to be consistent with previous NMR data reported for this compound (Li et al., 2012) :^1^H NMR (DMSO-*d6*, 400 MHz) *δ* 12.40 (1H, brs), 5.44 (1H, s), 4.31 (1H, d, *J*= 5.1), 3.01 (1H, td, *J*=11.4, 5.1), 2.85 (1H, dd, *J*= 13.7, 4.1), 2.60 (1H, dt, *J*= 13.4, 3.3), 2.30 (1H, s), 2.14-0.69 (18H, m) 1.34 (3H, s), 1.01 (3H, s), 0.91 (3H, s), 0.90 (3H, s), 0.89 (3H, s), 0.87 (3H, s), 0.68 (3H, s).

*Isolation and structural determination of 11β,16β-dihydroxy, 12,13β-epoxy-β-amyrin, 11-oxo,16β-hydroxy-β-amyrin and 11-oxo,12,13β-epoxy,16β-hydroxy-β-amyrin.*

Dry leaf powder (Table S2) was extracted with EtOAc via pressurized solvent extraction and then treated with Ambersep 900 OH. The crude extract was subjected to flash chromatography (120 g SiliaSep cartridge (Silicycle): Solvent A: [Hexane] Solvent B: [EtOAc]; gradient: 0% [B] to 100% [B] over 3000 mL; flow rate: 100 mL/min, 250 mL fractions). Fractions containing the compounds of interest were identified by GC-MS. Fraction 6 contained 11β,16β-dihydroxy, 12,13β-epoxy-β-amyrin and 11-oxo,16β-hydroxy-β-amyrin and fraction 7 contained 11-oxo,12,13β-epoxy,16β-hydroxy-β-amyrin. Fraction 6 was concentrated and treated with decolorizing charcoal before being subjected to additional fractionation by flash chromatography (using 2x10 g SnapUltra silica cartridge (Biotage) stacked together: Solvent A: [Hexane] Solvent B: [EtOAc]; gradient: 0 % [B] to 60 % [B] over 1500 mL; flow rate: 36 mL/min, 22 mL fractions). No significant separation of 11β,16β-dihydroxy,12,13β-epoxy-β-amyrin and 11-oxo,16β-hydroxy-β-amyrin was observed. Thus fractions containing these two compounds were combined, concentrated and subjected to additional fractionation by flash chromatography (2x10 g SnapUltra silica cartridge (Biotage) stacked together: Solvent A: [DCM] Solvent B: [EtOAc]; gradient: 0% [B] to 30% [B] over 3000 mL; flow rate: 36 mL/min, 22 mL fractions,). This afforded 6.0 mg of 11β,16β-dihydroxy, 12,13β-epoxy-β-amyrin and 3.5 mg of 11-oxo,16β-hydroxy-β-amyrin, both as white amorphous solids. Fraction 7 from the first fractionation, which contained 11-oxo,12,13β-epoxy,16β-hydroxy-β-amyrin, was concentrated and subjected to additional fractionation by flash chromatography (2x10 g SnapUltra silica cartridge (Biotage) stacked together: Solvent A: [Hexane] Solvent B: [EtOAc]; gradient: 0% [B] to 30% [B] over 3000 mL; flow rate: 36 mL/min, 22 mL fractions). Fractions containing 11-oxo,12,13β-epoxy,16β-hydroxy-β-amyrin were combined and concentrated and subjected to additional fractionation by flash chromatography (2x10 g SnapUltra silica cartridge (Biotage) stacked together: Solvent A: [DCM] Solvent B: [EtOAc]; gradient: 0% [B] to 30% [B] over 3000 mL; flow rate: 36 mL/min, 22 mL fractions). Fractions containing 11-oxo,12,13β-epoxy,16β-hydroxy-β-amyrin were combined, concentrated and treated with decolorizing charcoal. This afforded 14.0 mg of the compound as a white amorphous solid. The structures of each compound were determined by NMR. Assignments were made via a combination of ^1^H, ^13^C, DEPT-edited HSQC, HMBC and 2D NOESY experiments (Table S5-7).

*Isolation and structural determination of 12β-hydroxyoleanane-13β:28-olide*

Dry leaf powder (Table S2) was subject to pressurized solvent extraction in EtOAc. The extract was dried and subjected directly to fractionation by flash chromatography (120 g SiliaSep cartridge (Silicyle): Solvent A: [Hexane] Solvent B: [EtOAc]; gradient: 0% [B] to 100% [B] over 3000 mL; flow rate: 100 mL/min, 250 mL fractions. The fraction containing 12β-hydroxyoleanane-13β:28-olide (as assessed by GCMS) was concentrated and subjected to additional fractionation by flash chromatography (2x10 g SnapUltra silica cartridges (Biotage) stacked together: Solvent A: [hexane] Solvent B: [EtOAc]; gradient: 0% [B] to 100% [B] over 3080 mL; flow rate: 36 mL/min, 22 mL fractions). Fractions containing 12β-hydroxyoleanane-13β:28-olide (as assessed by GC-MS) were combined, concentrated and subjected to additional fractionation by flash chromatography (2x10 g SnapUltra silica cartridges (Biotage) stacked together: Solvent A: [DCM] Solvent B: [EtOAc]; gradient: 0% [B] to 60% [B] over 3080 mL; flow rate: 36 mL/min, 22 mL fractions). Fractions containing 12β-hydroxyoleanane-13β:28-olide (as assessed by GCMS) were combined, concentrated and subjected to additional fractionation by flash chromatography (10 g SnapUltra silica (Biotage): Solvent A: [DCM] Solvent B: [EtOAc]; gradient: 0 % [B] to 40 % [B] over 1500 mL; flow rate: 36 mL/min, 22 mL fractions). Fractions containing 12β-hydroxyoleanane-13β:28-olide (as assessed by GC-MS) were combined and concentrated affording 5.1 mg of product as a white amorphous solid. The structure was determined by NMR. Assignments were made via a combination of ^1^H, ^13^C, DEPT-edited HSQC, HMBC and 2D NOESY experiments (Table S8).

# Supplementary Figures

Figure S1. Mass spectra for the β-amyrin and EpHβA peaks shown in Figure 1.

**a.** Mass spectrum of the SAD1 product in crude *N. benthamiana* extracts at 11.17 min (top) versus that of a β-amyrin commercial standard (bottom) **b.** EI mass spectra of the CYP51H10 product as seen in crude *N. benthamiana* extracts at 12.99 min (top) versus that of the purified EpHβA standard (bottom). The structures of trimethylsilylated β-amyrin and EpHβA are shown inset on the mass spectra of the relevant standards.

Figure S2. Purity estimation of β-amyrin by HPLC-CAD

HPLC-CAD analysis of **a.** purified β–amyrin crystals and **b.** recovered mother liquor after flash chromatography. In both panels, the blank run (red) is overlaid with the experimental run (black).

Figure S3. Coexpression of individual CYPs with tHMGR and SAD1 in *N. benthamiana*

GC-MS total ion chromatograms of extracts from *N. benthamiana* leaves expressing single CYPs with *tHMGR* and *Sad1*. In each figure the *tHMGR/Sad1* control is shown in black with the CYP-expressing sample in red **a.** *CYP51H10* **b.** *CYP88D6* **c.** *CYP93E1* **d.** *CYP716A12* **e.** *CYP72A65.* The EI mass spectra for the oxidized forms of β-amyrin are shown on the right along with the structures.

Figure S4. Combinatorial biosynthesis using CYP93E1 and CYP716A12 in *N. benthamiana*.

GC-MS total ion chromatogram of extracts from *N. benthamiana* leaves (top). The *tHMGR/Sad1/CYP93E1/CYP716A12* sample is shown in black with single CYP controls in blue and orange. IS – Internal standard (coprostan-3-ol). Mass spectra for the combinatorial peaks (red arrows) are given below along with the structures. Structures shown in black (24-hydroxy-oleanolic acid and 24-aldehyde-oleanolic acid) match MS data from a previous study (Fukushima et al., 2013).

Figure S5. Combinatorial biosynthesis using CYP716A12 and CYP72A65 in *N. benthamiana*.

GC-MS total ion chromatograms of leaf extracts: *tHMGR/Sad1/CYP716A12/CYP72A65* is shown in black, single CYP controls in blue and orange. IS – internal standard (coprostan-3-ol). Mass spectra for these combinatorial peaks (red arrows) are shown below along with structures. The major product (30-hydroxy-oleanolic acid) matches MS data from previous studies (Burnouf-Radosevich et al., 1985; Fukushima et al., 2013).

Figure S6. Combinatorial biosynthesis using CYP88D6 and CYP72A65 in *N. benthamiana*.

GC-MS total ion chromatograms of leaf extracts: *tHMGR/Sad1/CYP88D6/CYP72A65* is shown in black, single CYP controls in blue and orange. IS – internal standard (coprostan-3-ol). The mass spectrum for the combinatorial peak (red arrow) is shown on the right with the expected structure. The spectrum for this compound (11-oxo,30-hydroxy-β-amyrin) matches MS data from a previous study (Seki et al., 2011).

Figure S7. Combinatorial biosynthesis using CYP93E1 and CYP72A65 in *N. benthamiana*.

GC-MS total ion chromatograms of leaf extracts: *tHMGR/Sad1/CYP93E1/CYP72A65,* is shown in black, single CYP controls in blue and orange. IS – internal standard (coprostan-3-ol). Mass spectra for these combinatorial peaks (red arrows) are shown below with expected structures. The spectrum for the major compound (24,30-dihydroxy-β-amyrin) matches MS data from a previous study (Fukushima et al., 2013), while those in grey (24-carboxy,30-hydroxy-β-amyrin and 24-carboxy,30-carboxy-β-amyrin) are putative structures inferred from MS data.

Figure S8. Combinatorial biosynthesis using CYP93E1 and CYP51H10 in *N. benthamiana*.

GC-MS total ion chromatogram of leaf extracts: *tHMGR/Sad1/CYP93E1/CYP51H10* is shown in black, single CYP controls in blue and orange. IS – internal standard (coprostan-3-ol). Mass spectra for the combinatorial peaks (red arrows) are given below with expected structures. The identity of the major product (12,13β-epoxy, 16β,24-dihydroxy-β-amyrin) shown in black was verified by NMR (Table S3). Structures shown in grey (12,13β-epoxy, 24-hydroxy-β-amyrin, 12,13β-epoxy,24-carboxy-β-amyrin and 12,13β-epoxy,16β-hydroxy,24-aldehyde-β-amyrin) are putative assignments based on the mass spectra.

Figure S9. Combinatorial biosynthesis using CYP93E1 and CYP88D6 in *N. benthamiana*.

GC-MS total ion chromatogram of leaf extracts: *tHMGR/Sad1/CYP93E1/CYP88D6* is shown in black, single CYP controls in blue and orange. IS – internal standard (coprostan-3-ol). Mass spectra for the combinatorial peaks (red arrows) are shown below with structures. The identity of the major product (11-oxo, 24-hydroxy-β-amyrin) shown in black was verified by NMR (Table S4). Structures shown in grey (11-oxo, 24-aldehyde-β-amyrin and 11-oxo, 24-carboxy-β-amyrin) are putative assignments based on the mass spectra.

Figure S10. Combinatorial biosynthesis using CYP716A12 and CYP88D6 in *N. benthamiana*.

GC-MS total ion chromatogram of leaf extracts: *tHMGR/Sad1/CYP716A12/CYP88D6* is shown in black, single CYP controls shown in blue and orange. IS – internal standard (coprostan-3-ol). Mass spectra for the combinatorial peaks (red arrows) are shown below with structures. The identity of the major compound (11-oxo-oleanolic acid) was verified by NMR. The structure shown in grey (11-oxo-erythrodiol) is a putative assignment based on the mass spectra.

Figure S11. Combinatorial biosynthesis using CYP88D6 and CYP51H10 in *N. benthamiana*.

GC-MS total ion chromatogram of leaf extracts: *tHMGR/Sad1/CYP88D6/CYP51H10* is shown in black, single CYP controls in blue and orange. IS – internal standard (coprostan-3-ol). Mass spectra for the combinatorial peaks (red arrows) are given below with structures. The identity of the compounds (11β,16β-dihydroxy,12,13β-epoxy-β-amyrin, 11-oxo,16β-hydroxy-β-amyrin and 11-oxo,12,13β-epoxy,16β-hydroxy-β-amyrin) were verified by NMR (Tables S5-7).

Figure S12. Combinatorial biosynthesis using CYP716A12 and CYP51H10 in *N. benthamiana*.

GC-MS total ion chromatogram of leaf extracts: *tHMGR/Sad1/CYP716A12/CYP51H10* is shown in black with single CYP controls in blue and orange. IS – internal standard (coprostan-3-ol). Mass spectra for the combinatorial peaks (red arrows) are given below with structures. The identity of the structure shown in black (12β-hydroxyoleanane-13β:28-olide) was verified by NMR (Table S8). The structure shown in grey (12β,16β-dihydroxyoleanane-13β:28-olide) is putatively assigned based on the mass spectrum.

Figure S13. Combinatorial biosynthesis using CYP72A65 and CYP51H10 in *N. benthamiana*.

GC-MS total ion chromatograms of leaf extracts: *tHMGR/Sad1/CYP72A65/CYP51H10* is shown in black, single CYP controls in blue and orange. IS – internal standard (coprostan-3-ol). The mass spectrum for the novel product (red arrow) is given below with the putative structure (12,13β-epoxy,16β,30-dihydroxy-β-amyrin) as inferred from MS data.

Figure S14. Major products generated by co-expression of CYPs with β-amyrin synthase in yeast

CYP93E9 is a functionally equivalent C-24 oxidase used in place of CYP93E1 (Moses et al., 2014c). Combinations of CYP51H10 with either CYP93E9, CYP88D6 or CYP716A12 in yeast resulted in products with different fragmentation patterns to the products observed in *N. benthamiana* that are likely to result from breakdown of the epoxide in the acid conditions of the yeast media. Ions shown in grey are background from a co-eluting peak in the GC-MS also found in controls.

| **Combination** | **Mass** | **RT** | **Identity** | **Assignment** |
| --- | --- | --- | --- | --- |
| *tHMGR/Sad1/ CYP93E1/ CYP716A12* | 688 | 13.5 | **24-hydroxy-oleanolic acid (4-epi-hederagenin) (#7)** | Matching MS (Fukushima et al., 2013) |
|  | 598 | 14.0 | Unknown | - |
|  | 614 | 15.3 | 24-aldehyde-oleanolic acid (4-epi-gypsogenin) | Matching MS (Fukushima et al., 2013) |
| *tHMGR/Sad1/ CYP716A12/ CYP72A65* | 688 | 14.9 | **30-hydroxy-oleanolic acid (queretaroic acid) (#8)** | Matching MS (Burnouf-Radosevich et al., 1985; Fukushima et al., 2013) |
|  | 688 | 15.1 | Unknown | - |
|  | 674 | 15.3 | Unknown | - |
| *tHMGR/Sad1/ CYP88D6/ CYP72A65* | 600 | 15.5 | **11-oxo,30-hydroxy-β-amyrin (#9)** | Matching MS  (Seki et al., 2011) |
| *tHMGR/Sad1/ CYP93E1/ CYP72A65* | 674 | 14.3 | **24,30-dihydroxy-β-amyrin (#10)** | Matching MS (Fukushima et al., 2013) |
|  | 688 | 15.6 | Unknown | - |
|  | 688 | 15.8 | 24-carboxy,30-hydroxy-β-amyrin | Inferred from MS |
|  | 702 | 16.7 | 24-carboxy,30-carboxy-β-amyrin | Inferred from MS |
| *tHMGR/Sad1/ CYP93E1/ CYP51H10* | 762 | 12.7 | Unknown | - |
|  | 688 | 13.2 | Unknown | - |
|  | 602 | 13.4 | 12,13β-epoxy,24-hydroxy-β-amyrin | Inferred from MS |
|  | 690 | 14.0 | **12,13β-epoxy,16β,24-dihydroxy-β-amyrin (#11)** | NMR^b^ |
|  | 688 | 14.2 | Unknown | - |
|  | 616 | 14.6 | 12,13β-epoxy,24-carboxy-β-amyrin | Inferred from MS |
|  | 690 | 14.8 | Unknown | - |
|  | 616 | 15.7 | 12,13β-epoxy,16β-hydroxy,24-aldehyde-β-amyrin | Inferred from MS |
| *tHMGR/Sad1/ CYP93E1/ CYP88D6* | 600 | 13.7 | Unknown | - |
|  | 600 | 14.2 | **11-oxo, 24-hydroxy-β-amyrin (#12)** | NMR^b^ |
|  | 526 | 15.5 | 11-oxo, 24-aldehyde-β-amyrin | Inferred from MS |
|  | 614 | 15.7 | 11-oxo, 24-carboxy-β-amyrin | Inferred from MS |
| *tHMGR/Sad1/ CYP716A12/ CYP88D6* | 686 | 12.2 | Unknown | - |
|  | 614 | 14.0 | Unknown | - |
|  | 600 | 14.3 | 11-oxo-erythrodiol | Inferred from MS |
|  | 614 | 15.2 | **11-oxo-oleanolic acid (#13)** | NMR^a^ |
| *tHMGR/Sad1/ CYP88D6/ CYP51H10* | 688 | 11.6 | Unknown | - |
|  | 690 | 12.1 | **11β,16β-dihydroxy, 12,13β-epoxy-β-amyrin (#14)** | NMR^b^ |
|  | 600 | 13.4 | 11-oxo,16β-hydroxy-β-amyrin (#15) | NMR^a^ |
|  | 616 | 13.9 | 11-oxo,12,13β-epoxy,16β-hydroxy-β-amyrin (#16) | NMR^b^ |
| *tHMGR/Sad1/ CYP716A12/ CYP51H10* | 598 | 12.5 | Unknown | - |
|  | 688 | 12.9 | **Unknown** | - |
|  | 602 | 13.3 | Unknown | - |
|  | 614 | 14.9 | Unknown | - |
|  | 615 | 15.0 | Unknown | - |
|  | 616 | 15.1 | Unknown | - |
|  | 616 | 16.1 | 12β-hydroxyoleanane-13β:28-olide (#17) | NMR^b^ |
|  | 601 | 16.2 | Unknown | - |
|  | 704 | 16.6 | 12β,16β-dihydroxyoleanane-13β:28-olide | Inferred from MS |
| *tHMGR/Sad1/ CYP51H10/ CYP72A65* | 690 | 15.2 | 12,13β-epoxy,16β,30-dihydroxy-β-amyrin | Inferred from MS |

Table S1. (above) Products generated by combinatorial biosynthesis in *N. benthamiana*.

Combinatorial biosynthetic compounds produced in *N. benthamiana* (see Figure S4-13 and Tables S2-8 for further information). The most abundant of the new products for each combination is indicated in bold. Numbering of compounds corresponds to the numbers in Fig. 3. RT, retention time. For NMR-assigned compounds, ^a^ indicates compounds that are represented in chemical search databases (Reaxys, SciFinder), and ^b^ indicates those compounds that were not present in these databases.

| **Constructs** | **Identity** | **No of**  **plants** | **Dry weight (g)** | **Amount purified (mg)** | **Yield (mg/g dw)** |
| --- | --- | --- | --- | --- | --- |
| *tHMGR/Sad1* | β-amyrin | 459 | 244 | 809 | 3.3 |
| *tHMGR/Sad1/CYP51H10* | 12,13β-epoxy,16β-hydroxy-β-amyrin | 70 | 20.4 | 79 | 3.87 |
|  |  |  |  |  |  |
| *tHMGR/Sad1/CYP88D6* | 11α-hydroxy-β-amyrin | 23 | 10.4 | 7 | 0.67 |
|  | 11-oxo-β-amyrin | 23 | 10.4 | 10.4 | 1 |
| *tHMGR/Sad1/CYP93E1* | 24-hydroxy-β-amyrin | 17 | 3 | 4.7 | 1.56 |
|  |  |  |  |  |  |
| *tHMGR/Sad1/CYP72A65* | 30-hydroxy-β-amyrin | 30 | 19 | 6.5 | 0.34 |
|  |  |  |  |  |  |
| *tHMGR/Sad1/ CYP93E1/ CYP51H10* | 12,13β-epoxy,16β,24-dihydroxy-β-amyrin | 96 | 30.2 | 17 | 0.56 |
|  |  |  |  |  |  |
|  |  |  |  |  |  |
| *tHMGR/Sad1/ CYP88D6/ CYP93E1* | 11-oxo, 24-hydroxy-β-amyrin | 112 | 18.5 | 41 | 2.2 |
|  |  |  |  |  |  |
|  |  |  |  |  |  |
| *tHMGR/Sad1/ CYP716A12/ CYP88D6* | 11-oxo-oleanolic acid | 141 | 70 | 46 | 0.66 |
|  |  |  |  |  |  |
|  |  |  |  |  |  |
| *tHMGR/Sad1/ CYP88D6/ CYP51H10* | 11β,16β-dihydroxy, 12,13β-epoxy-β-amyrin | 112 | 30.1 | 6 | 0.2 |
|  | 11-oxo,16β-hydroxy-β-amyrin | 112 | 30.1 | 3.5 | 0.12 |
|  | 11-oxo,12,13β-epoxy,16β-hydroxy-β-amyrin | 112 | 30.1 | 14 | 0.46 |
| *tHMGR/Sad1/ CYP51H10/ CYP716A12* | 12β-hydroxyoleanane-13β:28-olide | 96 | 29 | 5.1 | 0.18 |
|  |  |  |  |  |  |
|  |  |  |  |  |  |

Table S2. Compounds purified from *N. benthamiana* in this study.

| **Carbon numbering scheme and selected COSY and HMBC** | | | | | |
| --- | --- | --- | --- | --- | --- |
|  | | | | | |
| **Atom Number** | **^13^C δ** | **^1^H** | **Atom Number** | **^13^C δ** | **^1^H** |
| **3** | 80.55 | 3.62 (1H, dd, *J*=11.3, 4.3) | **21** | 35.09 | 1.58 (2H, m) |
| **13** | 67.56 | / | **29** | 33.81 | 0.95 (3H, s) |
| **16** | 66.09 | 4.56 (1H, dd, *J*=10.8, 4.6) | **22** | 31.90 | 2.39 (1H, dt, *J*=13.9, 3.5)  1.36 (1H, m) |
| **24** | 64.86 | 4.50 (1H, d, *J*=10.9)  3.72 (1H, d, *J*=10.9) | **20** | 31.35 | / |
| **5** | 56.93 | 0.92 (1H, m) | **2** | 28.83 | 2.00 (1H, m)  1.80 (1H, m) |
| **12** | 54.57 | 2.87 (1H, d, *J*=3.7) | **30** | 24.21 | 0.86 (3H, s) |
| **18** | 50.44 | 1.39 (1H, m) | **27** | 24.00 | 1.35 (3H, s) |
| **9** | 46.67 | 1.47 (1H, m) | **23** | 23.99 | 1.53 (3H, s) |
| **4** | 43.61 | / | **28** | 23.09 | 1.48 (3H, s) |
| **14** | 42.94 | / | **11** | 22.83 | 1.90 (1H, m)  1.80 (1H, m) |
| **8** | 42.23 | / | **26** | 21.14 | 1.32 (3H, s) |
| **19** | 42.05 | 1.89 (1H, m)  1.23 (1H, m) | **6** | 19.03 | 1.64 (1H, m)  1.49 (1H, m) |
| **17** | 39.68 | / | **25** | 16.21 | 0.89 (3H, s) |
| **1** | 38.75 | 1.51 (1H, m)  0.95 (1H, m) | **C3-OH*** | / | 5.45 (1H, brs) |
| **10** | 37.77 | / | **C16-OH*** | / | 5.78 (1H, brs) |
| **7** | 35.40 | 1.46 (1H, m)  1.20 (1H, m) | **C24-OH*** | / | 6.67 (1H, brs) |
| **15** | 35.30 | 2.30 (1H, m) |  |  |  |

Table S3. ^13^ C & ^1^H δ assignments for 12,13β-epoxy,16β,24-dihydroxy-β-amyrin.

Pyridine-*d5* [referenced to the most downfield residual solvent peak (^1^H δ 8.74, ^13^C δ 150.35)]..Coupling constants are reported as observed and not corrected for second order effects. Assignments were made via a combination of ^1^H, ^13^C, DEPT-edited HSQC, HMBC and 2D NOESY experiments. *may be interchangeable (OHs appearing as broad singlets in this sample, coupling to respective methine groups not observed in COSY). **NB:** primary alcohol was confirmed as being on the C24 position over the C23 position by 2D NOESY. An NOE was observed between the C24 methylene hydrogens and C25 methyl hydrogens. Where ^1^H resonances overlap δ is reported as the center of the HSQC cross peak.

| **Carbon numbering scheme and selected COSY and HMBC** | | | | | |
| --- | --- | --- | --- | --- | --- |
|  | | | | | |
| **Atom Number** | **^13^C δ** | **^1^H δ** | **Atom Number** | **^13^C δ** | **^1^H δ** |
| **11** | 200.03 | / | **7** | 33.75 | 1.64 (1H, m)  1.33 (1H, m) |
| **13** | 170.62 | / | **29** | 33.52 | 0.91 (3H, s) |
| **12** | 128.88 | 5.80 (1H, s) | **17** | 32.97 | / |
| **3** | 80.33 | 3.70 (1H, dt, *J*=11.8, 4.4) | **20** | 31.55 | / |
| **24** | 64.98 | 3.78 (1H, dd, *J*=10.5, 8.4)  4.78 (1H, dd, *J*=10.8, 1.9) | **28** | 29.21 | 0.85 (3H, s) |
| **9** | 62.67 | 2.54 (1H, s) | **2** | 28.94 | 2.22 (1H, m)  1.98 (1H, m) |
| **5** | 56.43 | 1.01 (1H, m) | **15** | 27.13 | 1.71 (1H, m)  1.07 (1H, m) |
| **18** | 48.17 | 2.14 (1H, m) | **16** | 26.96 | 2.01 (1H, m)  0.86 (1H, m) |
| **8** | 46.08 | / | **23** | 24.08 | 1.60 (3H, s) |
| **19** | 45.75 | 1.67 (1H, m)  0.99 (1H, m) | **30** | 23.95* | 0.87 (3H, s) |
| **14** | 44.09 | / | **27** | 23.95* | 1.36 (3H, s) |
| **4** | 44.02 | / | **26** | 19.17 | 1.13 (3H, s) |
| **1** | 40.08 | 3.23 (1H, dt, *J*=13.3, 3.5)  1.22 (1H, m) | **6** | 18.82 | 1.77 (1H, m)  1.52 (1H, m) |
| **10** | 37.86 | / | **25** | 17.79 | 1.36 (3H, s) |
| **22** | 37.18 | 1.42 (1H, m)  1.23 (1H, m) | **C3-OH** | / | 6.73 (1H, d, *J*=4.7) |
| **21** | 35.03 | 1.40 (1H, m)  1.11 (1H, m) | **C24-OH** | / | 5.39 (1H, dd, *J*=8.2, 2.2) |

Table S4. ^13^ C & ^1^H δ assignments for 11-oxo, 24-hydroxy-β-amyrin.

Pyridine-*d5* [referenced to the most downfield residual solvent peak (^1^H δ 8.74, ^13^C δ 150.35)]. Coupling constants are reported as observed and not corrected for second order effects. Assignments were made via a combination of ^1^H, ^13^C, DEPT-edited HSQC, HMBC and 2D NOSEY experiments. * this is **NOT** a typographical error C30 and C27 have the same δ. NB: primary alcohol was confirmed as being on the C24 position over the C23 position by 2D NOESY. An NOE was observed between the C24 methylene hydrogens and C25 methyl hydrogens. Where ^1^H resonances overlap δ is reported as the center of the HSQC cross peak.

| **Carbon numbering scheme and selected COSY and HMBC** | | | | | |
| --- | --- | --- | --- | --- | --- |
|  | | | | | |
| **Atom Number** | **^13^C δ** | **^1^H δ** | **Atom Number** | **^13^C δ** | **^1^H δ** |
| **3** | 78.83 | 3.54 (1H, dt, *J*=11.6, 4.3) | **21** | 35.05 | 1.56 (1H, m)  1.19 (1H, m) |
| **13** | 70.46 | / | **29** | 33.73 | 0.94 (3H, s) |
| **11** | 67.57 | 4.68 (1H, m) | **22** | 31.86 | 2.41 (1H, m)  1.39 (1H, m) |
| **16** | 66.01 | 4.57 (1H, dt, *J*=11.0, 5.4) | **20** | 31.34 | / |
| **12** | 59.82 | 3.24 (1H, d, *J*=3.8) | **23** | 29.53 | 1.27 (3H, s) |
| **5** | 57.18 | 0.89 (1H, dd, *J*=11.4, 1.9) | **2** | 28.64 | 2.12 (1H, m)  1.97 (1H, m) |
| **9** | 52.49 | 1.42 (1H, m) | **27** | 24.48 | 1.35 (3H, s) |
| **18** | 50.35 | 1.45 (1H, m) | **30** | 24.19 | 0.83 (3H, s) |
| **14** | 43.42 | / | **26** | 23.04 | 1.72 (3H, s) |
| **8** | 42.70 | / | **28** | 22.92 | 1.53 (3H, s) |
| **19** | 41.96 | 1.91 (1H m)  1.27 (1H m) | **6** | 19.10 | 1.66 (1H, m)  1.59 (1H, m) |
| **4** | 40.08 | / | **25** | 18.83 | 1.67 (3H, s) |
| **10** | 39.56 | / | **24** | 17.03 | 1.14 (3H, s) |
| **17** | 39.51 | / | **C3-OH** | / | 5.74 (1H, d, *J*=5.3) |
| **1** | 39.09 | 2.64 (1H, dt, *J*=12.8, 3.3)  1.46 (1H, m) | **C16-OH** | / | 5.82 (1H, d, J= 5.4) |
| **7** | 35.76 | 1.58 (1H, m)  1.26 (1H, m) | **C11-O­H** | / | 5.57 (1H, d, J= 8.2) |
| **15** | 35.63 | 2.39 (1H, m)  1.83 (1H, m) |  |  |  |
|  |  |  |  |  |  |

Table S5 ^13^C & ^1^H δ assignments for 11β,16β-dihydroxy, 12,13β-epoxy-β-amyrin.

**Pyridine-*d5*** [referenced to the most downfield residual solvent peak (^1^H δ 8.74, ^13^C δ 150.35)].Coupling constants are reported as observed and not corrected for second order effects. Assignments were made via a combination of ^1^H, ^13^C, DEPT-edited HSQC, HMBC and 2D NOESY experiments. NB: C16-OH was assigned as β, by 2D NOESY. No NOE was observed between the C11 hydrogen and C25 or C26 methyl hydrogens. NOEs were observed between the C11 hydrogen, and the C12 hydrogen, C9 hydrogen, and the C1 α Hydrogen. Where ^1^H resonances overlap δ is reported as the center of the HSQC cross peak.

| **Carbon numbering scheme and selected COSY and HMBC** | | | | | |
| --- | --- | --- | --- | --- | --- |
| **^^** | | | | | |
| **Atom Number** | **^13^C δ** | **^1^H δ** | **Atom Number** | **^13^C δ** | **^1^H δ** |
| **11** | 200.14 | / | **21** | 34.85 | 1.60 (1H, m)  1.20 (1H, m) |
| **13** | 169.64 | / | **29** | 33.59 | 0.93 (3H, s) |
| **12** | 128.81 | 5.89 (1H, s) | **7** | 33.55 | 1.72 (1H, m)  1.40 (1H, m) |
| **3** | 78.34 | 3.51 (1H, dt, *J*= 11.7, 5.0) | **20** | 31.45 | / |
| **16** | 64.08 | 4.57 (1H, dt, *J*= 11.2, 5.1) | **22** | 31.17 | 2.42 (1H, m)  1.30 (1H, m) |
| **9** | 62.01 | 2.51 (1H, s) | **23** | 29.20 | 1.28 (3H, s) |
| **5** | 55.81 | 0.89 (1H, m) | **2** | 28.67 | 2.06 (1H, m)  1.89 (1H, m) |
| **18** | 50.16 | 2.46 (1H, m) | **27** | 25.11 | 1.52 (3H, s) |
| **8** | 46.42 | / | **30** | 24.31 | 0.95 (3H, s) |
| **14** | 46.07 | / | **28** | 23.26 | 1.12 (3H, s) |
| **19** | 45.77 | 1.84 (1H, m)  1.08 (1H, m) | **26** | 19.41 | 1.25 (3H, s) |
| **1** | 40.28* | 3.24 (1H, m)  1.24 (1H, m) | **6** | 18.41 | 1.60 (1H, m)  1.45 (1H, m) |
| **4** | 40.28* | / | **25** | 17.35 | 1.38 (3H, s) |
| **17** | 38.26 | / | **24** | 17.03 | 1.11 (3H, s) |
| **10** | 38.10 | / | **C3-OH** | / | 5.78 (1H, d, *J*=5.4) |
| **15** | 36.93 | 2.08 (1H, m)  1.75 (1H, m) | **C16-OH** | / | 5.97 (1H, d, *J*=5.2) |
|  |  |  |  |  |  |

Table S6. ^13^ C & ^1^H δ assignments for 11-oxo,16β-hydroxy-β-amyrin.

**Pyridine-*d5*** [referenced to the most downfield residual solvent peak (^1^H δ 8.74, ^13^C δ 150.35)]. * this is **NOT** a typographical error C1 and C4 have the same δ. Coupling constants are reported as observed and not corrected for second order effects. Assignments were made via a combination of ^1^H, ^13^C, DEPT-edited HSQC, HMBC and 2D NOESY experiments. Where ^1^H resonances overlap δ is reported as the center of the HSQC cross peak.

| **Carbon numbering scheme and selected COSY and HMBC** | | | | | |
| --- | --- | --- | --- | --- | --- |
| **^^** | | | | | |
| **Atom Number** | **^13^C δ** | **^1^H δ** | **Atom Number** | **^13^C δ** | **^1^H δ** |
| **11** | 205.52 | / | **21** | 34.90 | 1.53 (1H m)  1.18 (1H, m) |
| **3** | 78.14 | 3.45 (1H, td, *J*=11.6, 4.8) | **7** | 34.55 | 1.69 (1H, m) |
| **13** | 69.85 | / | **29** | 33.63 | 0.94 (3H, s) |
| **16** | 65.71 | 4.60 (1H, td, *J*=11.0, 5.0) | **22** | 31.65 | 2.40 (1H, m)  1.37 (1H, m) |
| **9** | 63.65 | 2.30 (1H, s) | **20** | 31.27 | / |
| **12** | 59.03 | 3.15 (1H, s) | **23** | 29.20 | 1.24 (3H, s) |
| **5** | 55.57 | 0.83 (1H, m) | **2** | 28.64 | 2.00 (1H, m)  1.89 (1H, m) |
| **18** | 49.28 | 1.41 (1H, m) | **26** | 24.33 | 1.46 (3H, s) |
| **14** | 43.31 | / | **30** | 24.02 | 0.82 (3H, s) |
| **8** | 42.73 | / | **27** | 23.55 | 1.52 (3H, s) |
| **19** | 41.44 | 1.88 (1H, m)  1.18 (1H, m) | **28** | 22.89 | 1.43 (3H, s) |
| **4** | 40.30 | / | **6** | 18.13 | 1.56 (2H, m) |
| **1** | 39.68 | 3.18 (1H, dt, J=13.5,3.5)  1.25 (m) | **25** | 16.94 | 1.27 (3H, s) |
| **17** | 39.44 | / | **24** | 16.85 | 1.08 (3H, s) |
| **10** | 38.85 | / | **C3-OH** | / | 5.73 (1H, d, *J*=5.3) |
| **15** | 35.44 | 2.34 (1H, m)  1.90 (1H, m) | **C16-OH** | / | 6.00 (1H, d, J=5.5) |
|  |  |  |  |  |  |

Table S7. ^13^ C & ^1^H δ assignments for 11-oxo,12,13β-epoxy,16β-hydroxy-β-amyrin.

**Pyridine-*d5*** [referenced to the most downfield residual solvent peak (^1^H δ 8.74, ^13^C δ 150.35)]. Coupling constants are reported as observed and not corrected for second order effects. Assignments were made via a combination of ^1^H, ^13^C, DEPT-edited HSQC, HMBC and 2D NOESY experiments. Where ^1^H resonances overlap δ is reported as the center of the HSQC cross peak.

| **Carbon numbering scheme and selected COSY and HMBC** | | | | | |
| --- | --- | --- | --- | --- | --- |
|  | | | | | |
|  | | | | | |
|  | | | | | |
| **Atom Number** | **^13^C δ** | **^1^H δ** | **Atom Number** | **^13^C δ** | **^1^H δ** |
|  |  |  |  |  |  |
| **28** | 180.49 | / | **29** | 33.70 | 0.94 (3H, s) |
| **13** | 94.09 | / | **17** | 32.21 | / |
| **3** | 78.42 | 3.45 (1H, brm) | **11** | 29.64 | 2.12 (1H, m)  1.98 (1H, m) |
| **12** | 67.32 | 4.16 (1H, brm) | **23** | 29.07 | 1.25 (3H, s) |
| **5** | 55.89 | 0.81 (1H, m) | **2** | 28.69 | 1.85 (2H, m) |
| **9** | 50.01 | 1.39 (1H, m) | **22** | 28.66 | 1.77 (2H, m) |
| **10** | 44.59 | / | **15** | 28.21 | 2.04 (1H, m)  1.19 (1H, m) |
| **14** | 43.31 | / | **30** | 24.40 | 0.87 (3H, s) |
| **8** | 43.29 | / | **16** | 21.66 | 2.14 (1H, m)  1.32 (1H, m) |
| **18** | 43.18 | 3.23 (1H, dd, *J*=13.7, 2.2) | **27** | 19.85 | 1.17 (3H, s) |
| **4** | 39.97 | / | **26** | 19.34 | 1.33 (3H, s) |
| **1** | 39.77 | 1.74 (1H, m)  1.00 (1H, m) | **6** | 18.63 | 1.56 (1H, m) |
| **19** | 37.70 | 1.88 (1H, m)  1.56 (1H, m) | **25** | 16.90 | 0.86 (3H, s) |
| **20** | 37.55 | / | **24** | 16.71 | 1.03 (3H, s) |
| **21** | 35.02 | 1.40 (1H, m)  1.17 (1H, m) | **C3-OH** | / | 5.27 (1H, d, *J*=4.1) |
| **7** | 34.26 | 1.50 (1H, m)  1.21 (1H, m) | **C12-OH** | / | 6.35 (1H, d, *J*=8.6) |

Table S8. ^13^ C & ^1^H δ assignments for 12β-hydroxyoleanane-13β:28-olide.

**Pyridine-*d5*** [referenced to the most downfield residual solvent peak (^1^H δ 8.74, ^13^C δ 150.35)]. Coupling constants are reported as observed and not corrected for second order effects. Assignments were made via a combination of ^1^H, ^13^C, DEPT-edited HSQC, HMBC and 2D NOESY experiments. NB: C12-OH was assigned as β, by 2D NOESY and mechanistic reasoning. Primarily this was due to an NOE observed between the C12 hydrogen and C27 methyl hydrogens, and the lack of an observed NOE between the C12 hydrogen and C26 methyl hydrogens. Where ^1^H resonances overlap δ is reported as the center of the HSQC cross peak.

| **Strain** | **pESC-URA tHMG1** | **pAG423GAL** | **pAG425GAL** |
| --- | --- | --- | --- |
| 1 | GgbAS T2A MTR1 | EV | EV |
| 2 | GgbAS T2A MTR1 | CYP88D6 | EV |
| 3 | GgbAS T2A MTR1 | CYP51H10 | EV |
| 4 | GgbAS T2A MTR1 | CYP93E9 | EV |
| 5 | GgbAS T2A MTR1 | CYP716A12 | EV |
| 6 | GgbAS T2A MTR1 | CYP72A65 | EV |
| 7 | GgbAS T2A MTR1 | CYP51H10 | CYP88D6 |
| 8 | GgbAS T2A MTR1 | CYP93E9 | CYP88D6 |
| 9 | GgbAS T2A MTR1 | CYP716A12 | CYP88D6 |
| 10 | GgbAS T2A MTR1 | CYP72A65 | CYP88D6 |
| 11 | GgbAS T2A MTR1 | CYP93E9 | CYP51H10 |
| 12 | GgbAS T2A MTR1 | CYP716A12 | CYP51H10 |
| 13 | GgbAS T2A MTR1 | CYP72A65 | CYP51H10 |
| 14 | GgbAS T2A MTR1 | CYP716A12 | CYP93E9 |
| 15 | GgbAS T2A MTR1 | CYP72A65 | CYP93E9 |
| 16 | GgbAS T2A MTR1 | CYP72A65 | CYP716A12 |
|  |  |  |  |

Table S9. Yeast strains used in the present study.

Yeast strains expressing various CYP combinations were generated as described in materials and methods. EV, empty vector.

| **Name** | **Sequence** |
| --- | --- |
| P1 | CACCATGGCGCCCGAGAAAATGCCCGAG |
| P2 | TCMGCAGGCGATCTTGGACATGTCC |
| P3 | ATGGGGGCGCTGTCG |
| P4 | TCACTTGGCGTACAGTACACC |
| P5 | ATGGCTGAGGTCGCCGCC |
| P6 | TTAGAACTCCGCTTCCGGAGG |
| P7 | ATGGCGGCGGCAGCGGTGAAC |
| P8 | CTACTTCTGCCTCTTGTAGATC |
| P9 | GGGGACAAGTTTGTACAAAAAAGCAGGCTTA ATGGCGGCGGCAGCGGTG |
| P10 | GGGGACCACTTTGTACAAGAAAGCTGGGTA CTACTTCTGCCTCTTGTAG |
| P11 | GGGGACAAGTTTGTACAAAAAAGCAGGCTTA ATGGGGGCGCTGTCGCGG |
| P12 | GGGGACCACTTTGTACAAGAAAGCTGGGTA TCACTTGGCGTACAGTACACC |
| P13 | GGGGACAAGTTTGTACAAAAAAGCAGGCTTA ATGGCTGAGGTCGCCGCC |
| P14 | GGGGACCACTTTGTACAAGAAAGCTGGGTA TTAGAACTCCGCTTCCGGAGG |
| P15 | GGGGACAAGTTTGTACAAAAAAGCAGGCTTA ATGTGGAGGCTAACAATAGGTG |
| P16 | GGGGACCACTTTGTACAAGAAAGCTGGGTA TCAGCTCTTAATCGCAAGAAGTCG |
| P17 | GGGGACAAGTTTGTACAAAAAAGCAGGCT ATGGACATGACAATTTGCGTCGT |
| P18 | GGGGACCACTTTGTACAAGAAAGCTGGGT TTAGTTTGCAGGCATACGACATC |
| P19 | GGGGACAAGTTTGTACAAAAAAGCAGGCTTAA TGGAAGTGTTTATGTTT |
| P20 | GGGGACCACTTTGTACAAGAAAGCTGGGTA TTACAGTT TATGCAAAAT |
|  |  |
|  |  |

Table S10. List of primers

Primers used for gene cloning for generating expression constructs in *N. benthamiana* and yeast described in this study.

| **Compound** | **Class** | **Quoted yield #** | **Converted Yield ǂ** | **Total isolated** | **Ref** |
| --- | --- | --- | --- | --- | --- |
| Geraniol | Monoterpene | 27 µg/g FW | 0.27 mg/g DW | - | Vasilev et al., 2014 |
| Geraniol | Monoterpene | 129 µg/g FW | 1.29 mg/g DW | - | Dong et al., 2016 |
| Geraniol | Monoterpene | 93 μg/g FW | 0.93 mg/g DW | - | Fischer et al., 2015 |
| Amorphadiene | Sesquiterpene | 6.2 mg/kg FW | 0.06 mg/g DW | - | van Herpen et al., 2010 |
| Artemisinic acid diglucoside | Sesquiterpene | 39.5 mg/kg FW* | 0.40 mg/g DW | ** | van Herpen et al., 2010 |
| Costunolide | Sesquiterpene | 60 ng/g FW | 0.6 μg/g DW | - | Liu et al., 2011 |
| Valencene | Sesquiterpene | 0.70 µg/g/24h | - | - | Cankar et al., 2015 |
| Casbene | Diterpene | 227 ng/cm^2^ | - | - | Brückner and Tissier, 2013 |
| Cembratrienol | Diterpene | 2500 ng/cm^2^ | - | - | Brückner and Tissier, 2013 |
| Isopimaric acid | Diterpene | 55 μg/g DW | 0.06 mg/g DW | - | Gnanasekaran et al., 2015 |
| Levopimaradiene | Diterpene | 100 ng/cm^2^ | - | - | Brückner and Tissier, 2013 |
| 13*R*-(+)-Manoyl oxide | Diterpene | ~27 μg/g FW | 270 μg/g DW | - | Andersen-Ranberg et al. 2016 |
| Various diterpenes | Diterpene | - | - | 0.5-5 mg*** | Andersen-Ranberg et al. 2016 |
| EpHβA | Triterpene | - | **1.18 mg/g DW** | 20 mg | Geisler et al., 2013 |
| β-Amyrin | Triterpene | - | **3.32 mg/g DW** | 809 mg | Present study |

Table S11. Literature comparisons of reported terpene yields through transient expression in *N. benthamiana*

Abbreviations: FW, fresh weight; DW, dry weight. Quoted yields represent the highest average yield reported in the respective study. * The glycosylation of artemisinic acid results from endogenous *N. benthamiana* activity and corresponds to 16.6mg/kg FW free artemisinic acid. ** The authors of this study report purification of this compound for NMR analysis, however an isolated yield is not provided. ǂ In our experience, dried *N. benthamiana* leaf material has a mass of approximately 10% that of wet material, therefore conversions from fresh to dry weight were made by multiplying the quoted fresh weight yield by ten. *** Exact yields for each compound are not reported. # Values shown for mono-, sesqui- and di-terpenes are mostly theoretical yields derived from estimates of levels of the compounds in leaf extracts as assessed by GC or LC, since none of these compounds were purified. Those for the triterpenes (shown in bold) were calculated based on the amount of purified compound recovered from a known dry weight of leaf material.

**Supplementary References**

Brückner, K., Tissier, A., 2013. High-level diterpene production by transient expression in Nicotiana benthamiana. Plant methods. 9, 46.

Burnouf-Radosevich, M., Delfel, N. E., England, R., 1985. Gas chromatography-mass spectrometry of oleanane- and ursane-type triterpenes—application to Chenopodium quinoa triterpenes. Phytochemistry. 24**,** 2063-2066.

Cankar, K., Jongedijk, E., Klompmaker, M., Majdic, T., Mumm, R., Bouwmeester, H., Bosch, D., Beekwilder, J., 2015. (+)-Valencene production in Nicotiana benthamiana is increased by down-regulation of competing pathways. Biotechnol J. 10, 180-9.

Dong, L., Jongedijk, E., Bouwmeester, H., Van Der Krol, A., 2016. Monoterpene biosynthesis potential of plant subcellular compartments. The New phytologist. 209, 679-90.

Fischer, M. J., Meyer, S., Claudel, P., Perrin, M., Ginglinger, J. F., Gertz, C., Masson, J. E., Werck-Reinhardt, D., Hugueney, P., Karst, F., 2013. Specificity of Ocimum basilicum geraniol synthase modified by its expression in different heterologous systems. J Biotechnol. 163, 24-9.

Gnanasekaran, T., Vavitsas, K., Andersen-Ranberg, J., Nielsen, A. Z., Olsen, C. E., Hamberger, B., Jensen, P. E., 2015. Heterologous expression of the isopimaric acid pathway in Nicotiana benthamiana and the effect of N-terminal modifications of the involved cytochrome P450 enzyme. J Biol Eng. 9, 24.

Li, H., Zou, H., Gao, L. X., Liu, T., Yang, F., Lj, J. Y., Li, J., Qiu, W. W., Tang, J., 2012. Synthesis and Biological Evaluation of Oleanolic Acid Derivatives as Novel Inhibitors of Protein Tyrosine Phosphatase 1b. Heterocycles. 85**,** 1117-1139.

Liu, Q., Majdi, M., Cankar, K., Goedbloed, M., Charnikhova, T., Verstappen, F. W., de Vos, R. C., Beekwilder, J., van der Krol, S., Bouwmeester, H. J., 2011. Reconstitution of the costunolide biosynthetic pathway in yeast and Nicotiana benthamiana. Plos One. 6, e23255.

Seki, H., Sawai, S., Ohyama, K., Mizutani, M., Ohnishi, T., Sudo, H., Fukushima, E. O., Akashi, T., Aoki, T., Saito, K., Muranaka, T., 2011. Triterpene functional genomics in licorice for identification of CYP72A154 involved in the biosynthesis of glycyrrhizin. Plant Cell. 23**,** 4112-23.

van Herpen, T. W., Cankar, K., Nogueira, M., Bosch, D., Bouwmeester, H. J., Beekwilder, J., 2010. Nicotiana benthamiana as a production platform for artemisinin precursors. Plos One. 5, e14222.

Vasilev, N., Schmitz, C., Dong, L., Ritala, A., Imseng, N., Häkkinen, S. T., van der Krol, S., Eibl, R., Oksman-Caldentey, K.-M., Bouwmeester, H., Fischer, R., Schillberg, S., 2014. Comparison of plant-based expression platforms for the heterologous production of geraniol. Plant Cell Tiss Organ Cult. 117, 373-80.
